# N-glycosylation of mannose receptor (CD206) regulates glycan binding by C-type lectin domains

**DOI:** 10.1016/j.jbc.2022.102591

**Published:** 2022-10-13

**Authors:** Kathrin Stavenhagen, Akul Y. Mehta, Lisa Laan, Chao Gao, Jamie Heimburg-Molinaro, Irma van Die, Richard D. Cummings

**Affiliations:** 1 Department of Surgery, Beth Israel Deaconess Medical Center, Harvard Medical School, Boston, Massachusetts, USA; 2 Department of Molecular Cell Biology and Immunology, Amsterdam UMC (VU Medical Center), Amsterdam, The Netherlands

**Keywords:** mannose receptor, glycosylation, glycobiology, mass spectrometry, lectin, N-linked glycosylation, O-linked glycosylation, glycan microarray, glycoproteomics, BSA, bovine serum albumin, CFG, Consortium for Functional Glycomics, CHO, Chinese hamster ovary, CTLD, C-type lectin domain, ER, endoplasmic reticulum, FA, formic acid, Fuc, fucose, HEK, human embryonic kidney, HI FBS, heat-inactivated fetal bovine serum, Man, mannose, MR, mannose receptor, PAA, polyacrylamide, PS, penicillin-streptomycin

## Abstract

The macrophage mannose receptor (MR, CD206) is a transmembrane endocytic lectin receptor, expressed in selected immune and endothelial cells, and is involved in immunity and maintaining homeostasis. Eight of the ten extracellular domains of the MR are C-type lectin domains (CTLDs) which mediate the binding of mannose, fucose, and GlcNAc in a calcium-dependent manner. Previous studies indicated that self-glycosylation of MR regulates its glycan binding. To further explore this structure–function relationship, we studied herein a recombinant version of mouse MR CTLD4-7 fused to human Fc-portion of IgG (MR-Fc). The construct was expressed in different glycosylation-mutant cell lines to study the influence of differential glycosylation on receptor glycan-binding properties. We conducted site-specific N- and O-glycosylation analysis and glycosylation site characterization using mass spectrometry by which several novel O-glycosylation sites were identified in mouse MR and confirmed in human full-length MR. This information guided experiments evaluating the receptor functionality by glycan microarray analysis in combination with glycan-modifying enzymes. Treatment of active MR-Fc with combinations of exoglycosidases, including neuraminidase and galactosidases, resulted in the loss of *trans*-binding (binding of MR CTLDs to non-MR glycans), due to unmasking of terminal, nonreducing GlcNAc in N-glycans of the MR CTLDs. Regalactosylation of N-glycans rescues mannose binding by MR-Fc. Our results indicate that glycans within the MR CTLDs act as a regulatory switch by masking and unmasking self-ligands, including terminal, nonreducing GlcNAc in N-glycans, which could control MR activity in a tissue- and cell-specific manner or which potentially affect bacterial pathogenesis in an immunomodulatory fashion.

The macrophage mannose receptor (MR, CD206) is a type 1 transmembrane endocytic lectin receptor and plays an essential role in immunity and homeostasis (1). It is largely expressed in macrophages, dendritic cells, as well as in some endothelial cells, where it constantly shuttles between the plasma membrane and the endosomal compartment to endocytose ligands in a clathrin-dependent manner (2).

The MR belongs to the MR family, including M-type phospholipase A2 receptor, DEC-205/gp200-MR6, and Endo180/uPARAP. The extracellular portion is composed of an N-terminal cysteine-rich domain, a fibronectin-type II domain, and 8 C-type lectin domains (CTLDs) (3). The MR cysteine-rich domain binds sulfated glycans (4, 5, 6) and the fibronectin-type II domain is known to bind collagen (7). Via the CTLDs, the receptor has been reported to bind mannose (Man), GlcNAc, fucose (Fuc), and glucose in a calcium-dependent manner (8, 9, 10, 11, 12). While the MR contains eight CTLDs, only CTLD4 has carbohydrate affinity when expressed as a single recombinant unit (8, 10). When expressed together with CTLD5, the CTLD4 binds mannose with the same affinity as all eight CTLDs together. For the recognition of multivalent ligands, such as yeast mannan, CTLD4-7/8 is required, indicating that the other CTLDs are important structural components (8, 10). Endogenous ligands of the MR CTLDs are mainly paucimannose and oligomannose N-glycans, which are present across a variety of different tissue types in control and cancer tissues (13).

The MR is a glycoprotein carrying seven potential N-glycosylation sites in mouse and eight in humans, and based on predictions using NetOGlyc (14), it may also contain several O-glycosylation sites, with four of them identified by sequence analysis (15). Specific MR glycosylation features have been reported to be necessary for ligand binding via the CTLDs (16) and previous studies have shown differential MR glycan signatures across different tissues (17), indicating tissue-specific functional regulation. Oddly, however, none of the N-glycosylation sites or predicted O-glycosylation sites are in CTLD4 or CTLD5, and thus, the glycosylation influence might be indirect. We recently reported that resident and elicited murine macrophages differ in sialylation and other features of N-glycosylation (18), and thus changes in glycosylation of the MR accompanying cellular differentiation could influence its function. Thus, it can be hypothesized that the biological function of the MR is cell- and tissue-dependent and could vary depending on environmental changes that influence glycosylation of the MR-bearing cells, such as inflammation or exposure to microbes (19). In order to enable the analysis of structure–function relationships of the MR, and learn more about its specific role in infection and immunity, we performed a glycoproteomic analysis of the MR in different glycosylation-mutant cell lines. During the process, we found that the contribution of glycosylation towards MR activity is more complex than previously described (16).

Here, we used a recombinant fusion protein composed of the MR CTLD4-7 and the IgG Fc portion (MR-Fc) to investigate the role of MR glycosylation in receptor-glycan binding. The MR-Fc was expressed in the Chinese hamster ovary (CHO) mutant cell lines Lec2 and PIR-P3, next to CHO WT and human embryonic kidney (HEK)293 WT cell lines. Differential MR-Fc binding to glycan microarrays was correlated with their site-specific N- and O-glycosylation. These studies revealed the critical glycan features of the MR-Fc that cause its loss or gain of external or *trans*-binding of glycans, that is, the binding of MR CTLDs to non-MR glycans. Consequently, enzymatic deglycosylation and reglycosylation allowed us to mimic this functional switch. Our data demonstrate that the masking (gain of *trans*-binding) or unmasking (loss of *trans*-binding) of nonreducing terminal GlcNAc residues in N-glycans regulate the glycan binding of the MR CTLDs.

## Results

### MR-Fc from CHO Lec2 and CHO PIR-P3 cell lines do not bind to glycan microarrays

**Figure 1:**
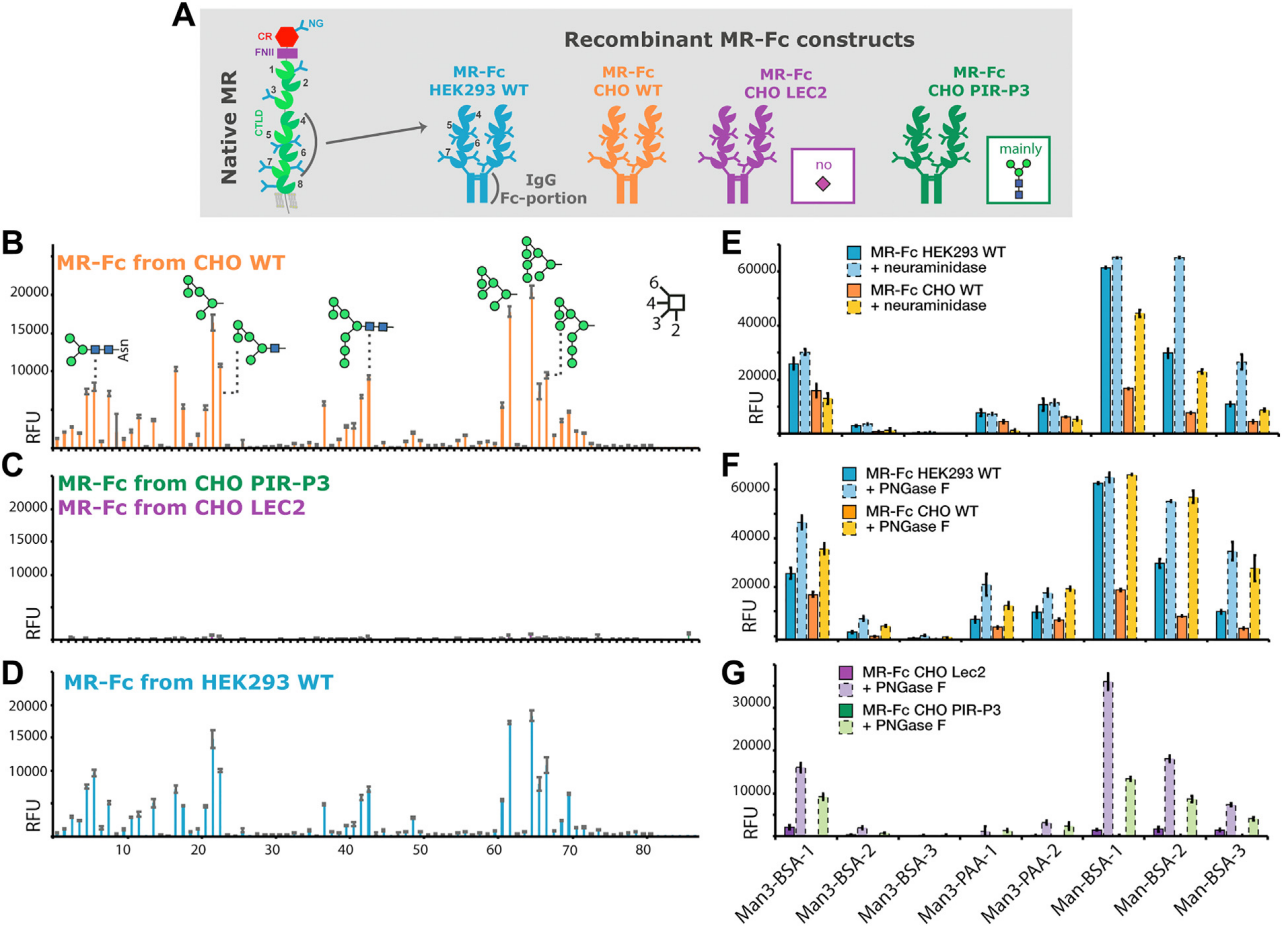
**Glycan microarray binding of MR-Fc constructs is regulated by N-glycosylation features.***A*, MR-Fc constructs comprising the murine CTLD4-7, fused to a human Fc-portion, were expressed in HEK293 WT, CHO WT, CHO Lec2 (reduced expression of sialic acid), and CHO PIR-P3 (mainly Man_3_GlcNAc_2_ N-glycans) cells. The oligomannose array was probed with (*B*) MR-Fc_CHO-WT_, (*C*) MR-Fc_Lec2_, MR-Fc_PIR-P3_, and (*D*) MR-Fc_HEK WT_, showing identical mannose binding of the two WT MR-Fcs and no binding for MR-Fc expressed in CHO Lec2 and CHO PIR-P3 cell lines. *E*, Man-BSA array was probed with MR-Fc_HEK-WT_ and MR-Fc_CHO-WT_ treated with neuraminidase. *F*, MR-Fc_HEK-WT_, MR-Fc_CHO-WT_, and (*G*) MR-Fc_Lec2_, MR-Fc_PIR-P3_ were treated with PNGase F for N-glycan release, resulting in gain of MR-Fc_Lec2_ and MR-Fc_PIR-P3_ mannose binding on Man-BSA array. Detection of all microarrays was performed using goat anti-human IgG Alexa-Fluor 488–conjugated at 5 μg/ml. Standard deviation is shown. Glycan linkages are indicated based on the Oxford system (38). *Green circle* = mannose; *blue square* = N-acetylglucosamine; Glycans on the array are listed in Tables S3 and S4. BSA, bovine serum albumin; CHO, Chinese hamster ovary; CTLD, C-type lectin domain; HEK, human embryonic kidney; Man, mannose; MR, mannose receptor; RFU, relative fluorescence units.

To explore the influence of glycosylation on MR binding to glycan ligands, we used the mouse CTLD4-7, fused to the Fc-portion of human IgG (MR-Fc). MR-Fc is a well-established probe to study the glycan ligands of the CTLDs (13). We expressed the MR-Fc in CHO cell mutants with different glycan features (Fig. 1*A*). CHO Lec2 cells (expressing MR-Fc_Lec2_) are characterized by a defect in their CMP–sialic acid transport (20), resulting in an overall reduction in glycoconjugate sialylation. The MR-Fc was also expressed in the CHO-PIR-P3 cell line (MR-Fc_PIR-P3_), which has a “PIR” mutation (resistance to glycoprotein processing inhibitors) and a defect in N-acetylglucosaminyltransferase I activity, resulting in mainly truncated paucimannose-type Man_3_GlcNAc_2_ N-glycans (21). As a reference, MR-Fc was also expressed in CHO WT (MR-Fc_CHO-WT_) as well as HEK293T WT cells (MR-Fc_HEK-WT_).

MR-Fc from all four cell lines were screened for their overall glycan binding using the Consortium for Functional Glycomics (CFG) glycan microarray (version 5.3) (Fig. S1 and Table S1). While the MR-Fc_CHO-WT_ binds to mainly paucimannose- and oligomannose-type glycans and glycan fragments on the array (Fig. S1*A*), MR-Fc_Lec2_ and MR-Fc_PIR-P3_ did not bind to the glycan array (Fig. S1, *C* and *D*) at the same concentrations. As the MR-Fc_CHO-WT_ mainly bound to mannose-terminating glycans, we also used it to probe our newly developed oligomannose array (Fig. 1*B* and Table S2). MR-Fc_CHO-WT_ bound to paucimannose and oligomannose N-glycans or truncated fragments from Man_3_ to Man_8_ on the oligomannose array. Consistent with the data from the CFG array, the MR-Fc_CHO-Lec2_ and MR-Fc_PIR-P3_ did not bind to the oligomannose array (Fig. 1*C*).

As the MR-Fc_CHO-WT_ and MR-Fc_HEK-WT_ exhibited identical binding patterns to the oligomannose array (Fig. 1, *B* and D) and CFG glycan array (Fig. S1, *A* and *B*), making them both suitable probes to study MR-Fc glycan binding, we prepared larger quantities of MR-Fc_HEK-WT_ to further explore the sites of glycosylation and roles of terminal glycan modifications. We developed a smaller subarray (Man-BSA array) to evaluate MR-Fc glycan-binding ability under different experimental conditions. The Man-bovine serum albumin (BSA) array contains different mannose conjugates, including Man_3_-BSA, Man-BSA, Man_3_-PAA, and their corresponding controls BSA, GlcNAc-BSA, and Glu-PAA (Table S3). The array was probed with MR-Fc_HEK-WT_, which bound to the mannose conjugates in a calcium-dependent manner as expected (Table S3).

### Desialylation of WT MR-Fc does not affect glycan binding

Proteins expressed in CHO Lec2 cells are deficient in sialic acid due to the deficient CMP–sialic acid transport. Thus, to simulate the MR-Fc_Lec2_ glycan phenotype, MR-Fc_HEK-WT_ was desialylated using neuraminidase. Desialylation was confirmed by lectin blots (Fig. S2*A*). MR-Fc_HEK-WT_ and the neuraminidase-treated MR-Fc_HEK-WT_ bound in a similar fashion to the Man-BSA array without loss of activity (Fig. 1*E* and Table S4). Next, both forms of MR-Fc_HEK-WT_ were enriched using mannan-agarose beads, showing no difference in mannan-agarose binding upon desialylation (Fig. S3). Thus, these results demonstrate that removal of sialic acid from a previously active MR-Fc does not result in loss of receptor *trans*-binding to mannose. This strongly indicates that the lack of glycan binding by MR-Fc_Lec2_ is independent of its sialylation status.

### N-glycan removal rescues glycan binding of MR-Fc_Lec2_ and MR-Fc_PIR-P3_

All four forms of MR-Fc were de–N-glycosylated using PNGase F (Fig. S2) to investigate the importance of N-glycans for MR-Fc mannose binding. For both MR-Fc_CHO-WT_ and MR-Fc_HEK-WT_, removal of N-glycans did not affect their positive binding to the Man-BSA array (Fig. 1*F* and Table S4). Unexpectedly, removal of the N-glycans from the MR-Fc_Lec2_ and MR-Fc_PIR-P3_ rescued their ability to bind to the Man-BSA array, in which case both treated receptors exhibited the same binding pattern as the WT MR-Fc (Fig. 1*G* and Table S4). This result indicates that N-glycosylation is not directly necessary for glycan binding by the MR-Fc but that when present, specific features of the N-glycans can inhibit the receptor functionality.

### N-glycosylation site characterization of MR-Fc reveals incomplete antennae galactosylation in MR-Fc_Lec2_

**Figure 2:**
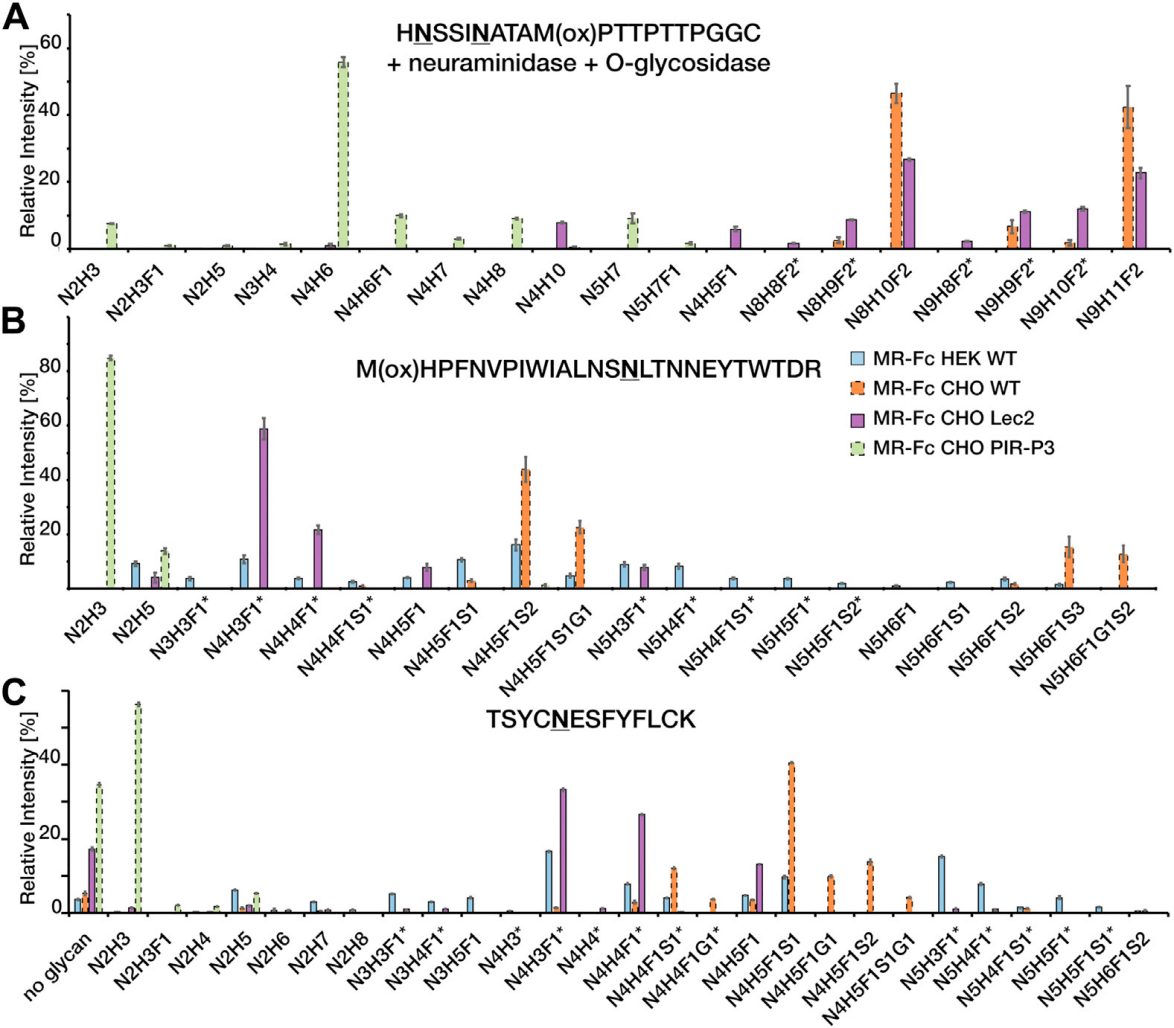
**N-glycosylation site characterization of MR-Fc expressed in HEK293 WT, CHO WT, CHO PIR-P3, and CHO Lec2 cells.** Relative quantitation of intact N-glycopeptides after tryptic in-gel digestion and LC-MS analysis. Peak intensities were extracted using LaCy-Tools (36), and relative quantitation was performed based on the total intensity of glycopeptides with the same peptide portion. *A*, tryptic N-glycopeptides around glycosylation sites Asn926 and Asn930. Samples were additionally treated with neuraminidase and O-glycosidase to reduce sample heterogeneity. *B*, tryptic glycopeptides around N-glycosylation site Asn1159 and (*C*) Asn1204. Experiments were performed in triplicates and the standard deviation is shown. N – N-acetylglucosamine, H – Hexose, F – Fucose, S – N-acetylneuraminic acid., and G – N-glycolylneuraminic acid. ∗ indicates glycoforms with a proposed terminal GlcNAc. CHO, Chinese hamster ovary; HEK, human embryonic kidney; MR, mannose receptor.

The MR-Fc has five predicted N-glycosylation sites: two in the linker region connecting CTLD5 and CTLD6 (Asn926 and Asn930, numbering based on complete MR sequence), two in CTLD7 (Asn1159 and Asn1204), and one N-glycan in the IgG Fc-portion. A detailed site-specific glycopeptide characterization using LC-MS analysis was performed to better understand the involvement of MR glycosylation in modulating its glycan binding (Fig. 2, Tables S5 and S6). The structural assignments were largely based on glycan composition.

Both glycosylation sites Asn926 and Asn930 are present on the same tryptic glycopeptide _925_HNSSINATAM(ox)PTTPTTPGGC_944_ (underlined Asn indicates glycosylation site). Since this region also contains O-glycosylation sites, as described below, the microheterogeneity was too complex to detect and quantify the glycopeptides under the chosen conditions. In an attempt to reduce glycopeptide complexity, the protein was treated with neuraminidase and O-glycosidase prior to in-gel trypsin treatment and glycopeptide analysis. This allowed N-glycopeptide analysis for all MR-Fc expressed in CHO cells, as the O-glycosidase efficiently removes core 1 O-glycans expressed by these cells (Fig. 2, *A*–*C*). For MR-Fc_HEK-WT_, N-glycosylation site characterization was not achieved, as HEK cells express more complex O-glycans, such as the core 2 O-glycans, which could not be released by O-glycosidase, and they also exhibit a higher degree of N-glycan microheterogeneity in general.

After neuraminidase treatment, the main composition of the doubly N-glycosylated peptide in MR-Fc_CHO-WT_ is Hex_10_HexNAc_8_Fuc_2_ (47%) and Hex_11_HexNAc_9_Fuc_2_ (42%), which can be most likely attributed to a combination of two Hex_5_HexNAc_4_Fuc_1_ *versus* one Hex_5_HexNAc_4_Fuc_1_ plus one Hex_6_HexNAc_5_Fuc_1_, respectively (Fig. 2*A*). These are also the main glycoforms in MR-Fc_Lec2_ (26% and 22%, respectively), indicating that the majority of glycans feature complete antennae galactosylation (Fig. 2*A*). However, the minor compositions such as Hex_(8-9)_HexNAc_8_Fuc_2_ (10%) and Hex_(8-9)_HexNAc_9_Fuc_2_ (13%) also indicate incomplete antenna galactosylation in MR-Fc_Lec2_. Smaller amounts of a singly occupied glycosylation site in this region were also observed, next to mannose-terminating glycans. MR-Fc_PIR-P3_ only contains mannose-terminating glycans, including the core fucosylated glycan Man_3_GlcNAc_2_Fuc_1_ (Fig. 2*A*). The glycopeptide with the composition Hex_7_HexNAc_5_ is most likely the result of incomplete O-glycosidase treatment, resulting in a glycopeptide with two Hex_3_HexNAc_2_ N-glycans and one Hex_1_HexNAc_1_ O-glycan. Smaller amounts of remaining O-glycans might also be present in this doubly N-glycosylated peptide in all analyzed MR-Fc.

The glycopeptides containing Asn1159 and Asn1204 in CTLD7 were characterized as individual glycopeptides in all four MR-Fc versions using trypsin treatment. Asn1159 was observed in the tryptic glycopeptide _1145_M(ox)HPFNVPIWIALNSNLTNNEYTWTDR_1170_. MR-Fc_CHO-WT_ contained almost exclusively biantennary di-sialylated (68%) and triantennary trisialylated (28%) core fucosylated N-glycans (Fig. 2*B*). MR-Fc_Lec2_ carried 59% glycans with Hex_3_HexNAc_4_Fuc_1_ and 8% Hex_3_HexNAc_5_Fuc_1_, accounting for ungalactosylated biantennary and triantennary N-glycans, respectively, suggesting incomplete antennae galactosylation (Fig. 2*B*). Another 22% of the peptides carry Hex_4_HexNAc_4_Fuc_1_, representing mono-galactosylated biantennary N-glycans and 8% Hex_5_HexNAc_4_Fuc_1_, the respective di-galactosylated version. Smaller amounts of Man_5_GlcNAc_2_ (4%) were also found. MR-Fc_PIR-P3_ contained only Man_3_GlcNAc_2_ and Man_5_GlcNAc_2_ linked to Asn1204 (Fig. 2*B*). Similar to the observations for Asn1159, MR-Fc_HEK-WT_ carried a larger variety of N-glycans attached to Asn1204, with 19% mono-sialylated and 26% di-sialylated, as well as 23% nongalactosylated, 18% mono-, 41% di-galactosylated, and 8% trigalactosylated N-glycans (Fig. 2*B*).

Asn1204 was detected in the tryptic peptide _1200_TSYCNESFYFLCK_1212_. MR-Fc_CHO-WT_ carries 67% mono-sialylated, with the major glycan Hex_5_HexNAc_4_Fuc_1_NeuAc_1_, and 18% di-sialylated biantennary N-glycans (Fig. 2*C*). MR-Fc_Lec2_ features only neutral N-glycans with 35% nongalactosylated, 29% mono-, and 13% di-galactosylated and biantennary N-glycans (Fig. 2*C*). A portion of this glycosylation site is not occupied with a glycan. Although signal intensities of glycopeptides and nonglycosylated peptides can feature different ionization efficiencies, they were still used for relative quantitation next to each other to enable the comparison of the four constructs. Man_3_GlcNAc_2_ and Man_5_GlcNAc_2_ were the main glycans observed attached to Asn1204 in MR-Fc_PIR-P3_ (Fig. 2*C*). MR-Fc_HEK-WT_ showed the most diverse microheterogeneity among all MR-Fcs, containing 22 different glycoforms with 21% mono-sialylated and 0.4% di-sialylated, as well as 37% nongalactosylated, 21% mono-, and 20% di-galactosylated N-glycans. In addition, smaller amounts (11%) of mannose-terminating N-glycans were detected (Fig. 2*C*). The N-glycosylation of the IgG Fc-portion contains typical IgG glycosylation for all MR-Fc except of MR-Fc_PIR-P3_, which again only carries mannose-terminating N-glycans (Fig. S4).

### Terminal GlcNAc-containing N-glycans result in the loss of MR-Fc mannose binding and can be restored by regalactosylation

**Figure 3:**
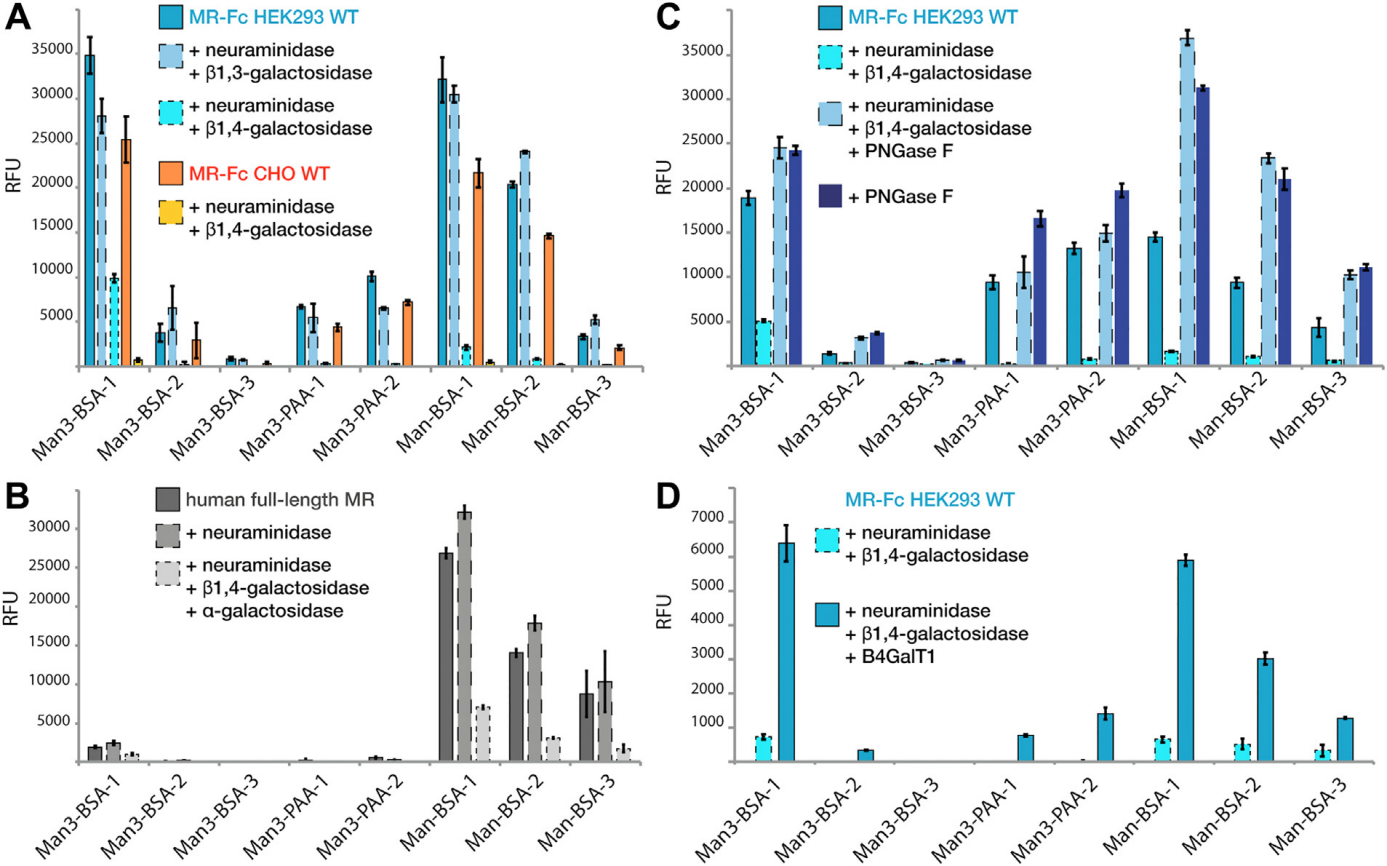
**Exposure and masking of terminal GlcNAc of MR N-glycans regulates receptor mannose binding on the Man-BSA array.***A*, MR-Fc_HEK-WT_ and MR-Fc_CHO-WT_ were treated with neuraminidase + β1,4-galactosidase and MR-Fc_HEK-WT_ also with β1,3-galactosidase, resulting in a major reduction in mannose binding on the Man-BSA array after removal of β1,4-linked mannose. *B*, human full-length MR was treated with neuraminidase and/or β1,4-galactosidase + α-galactosidase, confirming the reduced receptor binding to the Man-BSA array upon removal of terminal sialic acid and galactose and thus, exposure of terminal GlcNAc. *C*, MR-Fc_HEK-WT_ as well as degalactosylated and repurified MR-Fc_HEK-WT_ (reduced mannose binding) were treated with PNGase F for N-glycan removal. N-glycan removal of degal-MR-Fc_HEK-WT_ restores its mannose to a similar level as the control PNGase F–treated MR-Fc_HEK-WT_. *D*, degalactosylated and repurified MR-Fc_HEK-WT_ (reduced mannose binding) was enzymatically regalactosylated using β4GalT1, resulting in a partial restoration of mannose binding to the Man-BSA array. This indicates the influence of presenting self-ligands and the subsequent masking of these self-ligands as a regulatory feature to modulate MR mannose *trans*-binding. Detection of all microarrays was performed using goat anti-human IgG Alexa-Fluor 488–conjugated at 5 μg/ml for MR-Fc and anti-penta-His Alexa-Fluor 488 conjugate at 5 μg/ml for human full-length MR. Standard deviation is shown. BSA, bovine serum albumin; Man, mannose; MR, mannose receptor; RFU, relative fluorescence units.

Interestingly, across all MR N-glycosylation sites within MR-Fc_Lec2_, there is also a large portion of nongalactosylated or mono-galactosylated N-glycans present, in particular at the Asn1204 site (Fig. 2*C*), resulting in expression of MR-Fc_Lec2_ glycans with terminal, nonreducing GlcNAc. This was unexpected, as Lec2 CHO cells are only deficient in the CMP–sialic acid transport. However, GlcNAc is a reported ligand of the MR CTLDs (8, 9, 11, 12), and we confirmed that as we observed MR-Fc_HEK WT_ bind to the chitin-like oligosaccharide GlcNAc_3_-AEAB on the *Schistosoma mansoni* glycan array (13). However, binding to GlcNAc was not detected in any of our defined endogenous glycan microarrays or glycoprotein pull-down studies from mammalian cells (13). Furthermore, MR-Fc does not bind to the GlcNAc-BSA printed on the Man-BSA array (Table S3). Nevertheless, in order to more deeply evaluate if the terminal GlcNAc could present a potential self-ligand to the MR, thereby diminishing MR-Fc_Lec2_ mannose *trans*-binding, we modified the MR-Fc_HEK-WT_ and MR-Fc_CHO-WT_ by desialylation and degalactosylation with neuraminidase and β1,4-galactosidase, respectively, to simulate the MR-Fc_LEc2_ glycan phenotype. Both WT MR-Fcs were unable to bind to the Man-BSA array after removal of sialic acid and β1,4-galactose; as a control, treatment with neuraminidase and a β1,3-galactosidase had no effect (Fig. 3*A* and Table S7). Thus, removal of terminal sialic acids and β1,4-galactose residues results in the inactivation of MR-Fc. In addition, incubation of MR-Fc_HEK-WT_ with 100 mM mannose or GlcNAc, but not galactose, completely inhibited mannose binding (Fig. S5 and Table S8). We further found that the MR-Fc_HEK-WT_ can bind to GlcNAc-agarose beads and be specifically eluted with 100 mM GlcNAc or mannose, but not with galactose (Fig. S6), thus validating that GlcNAc is a ligand of the MR-Fc in this experimental approach. The N-glycopeptide analysis of MR-Fc_HEK WT_ also indicated the presence of under-galactosylated antennae (Fig. 2), which would result in a portion of inactive receptor.

Enzymatic removal of sialic acid and galactose was also performed on the commercial full-length recombinant human MR to exclude the possibility that the observed phenomenon is an artifact of the artificial presentation of the MR-Fc. Since the recombinant full-length MR was produced in murine cells, the N-glycans contain α-galactose; thus, next to treatment with β1,4-galactosidase, we did an additional treatment with α-galactosidase. These combined treatments resulted in reduced binding to the Man-BSA array (Fig. 3*B* and Table S7). Our results indicate that exposure of terminal, nonreducing GlcNAc residues diminishes the activity of the full-length MR, consistent with the results seen in the MR-Fc constructs.

**Figure 4:**
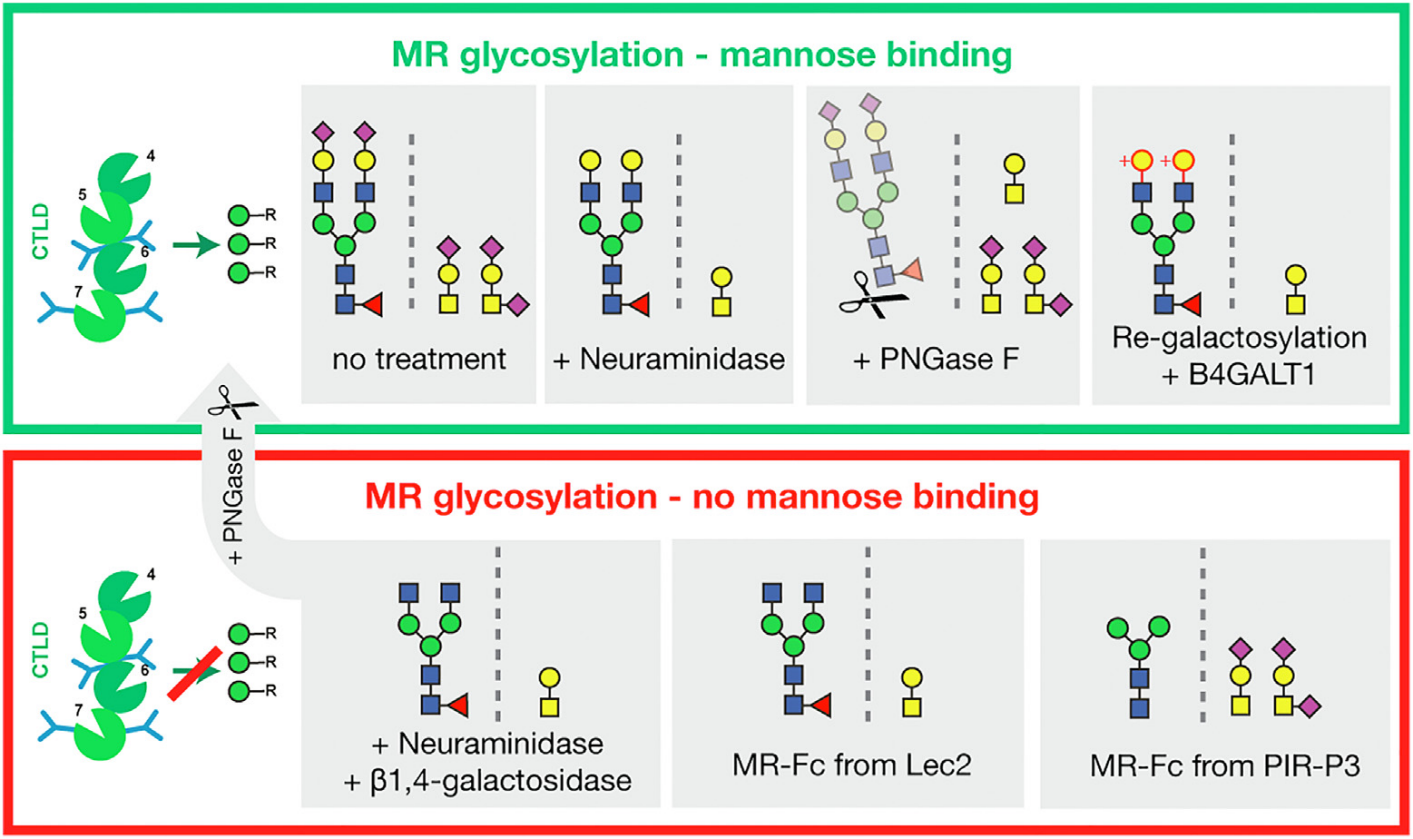
**Overview of the influence of MR-Fc glycosylation features on mannose *trans*-binding.** MR-Fc_HEK-WT_ and MR-Fc_CHO-WT_ bind mannose, which is not influenced by neuraminidase treatment or N-glycan removal by PNGase F. MR-Fc_Lec2_ and MR-Fc_PIR-P3_ from mutant cell lines are inactive and do not bind mannose due to presentation of self-ligands but can be rescued by N-glycan removal with PNGase F. In addition, the active MR-Fc_HEK-WT_ and MR-Fc_CHO-WT_ can be inactivated by neuraminidase and β1,4-galactosidase treatment, exposing terminal GlcNAc on the receptor N-glycans. In return, the inactive degal-MR-Fc_HEK-WT_ can partially regain mannose binding by regalactosylation and masking of the terminal GlcNAc. *Green circle* = mannose, *yellow circle* = galactose, *blue square* = N-acetylglucosamine, *yellow square* = N-acetylgalactosamine, *red triangle* = fucose, and *purple diamond* = N-acetylneuraminic acid. MR, mannose receptor.

Since degalactosylation can abolish MR and MR-Fc glycan binding, which would be predicted to expose GlcNAc residues, we investigated whether regalactosylation could rescue mannose binding of MR-Fc. MR-Fc_HEK-WT_ was first desialylated and degalactosylated, followed by protein A purification to remove residual glycosidases. This treatment resulted in loss of function, as demonstrated by reduced binding to the Man-BSA array (Fig. 3*C*). PNGase F treatment of the degalactosylated inactive degal-MR-Fc_HEK-WT_ rescued mannose binding and restored identical binding to the Man-BSA array, as observed for the PNGase F–treated active MR-Fc_HEK-WT_ (Fig. 3*C* and Table S7). Ultimately, regalactosylation of the inactive degal-MR-Fc_HEK-WT_ using β1,4-galactosyltransferase restored its binding to the Man-BSA array (Fig. 3*D*). The effectiveness of regalactosylation was also confirmed by lectin blots and binding of *Ricinus communis* agglutinin-I (Fig. S7). Overall, these results demonstrate that unmasked terminal GlcNAc on MR-Fc inactivates the receptor *trans*-binding to mannose and that regalactosylation, or the removal of all N-glycans, can restore mannose binding (summarized in Fig. 4).

### MR contains O-glycans in the linker region between the CTLDs

**Figure 5:**
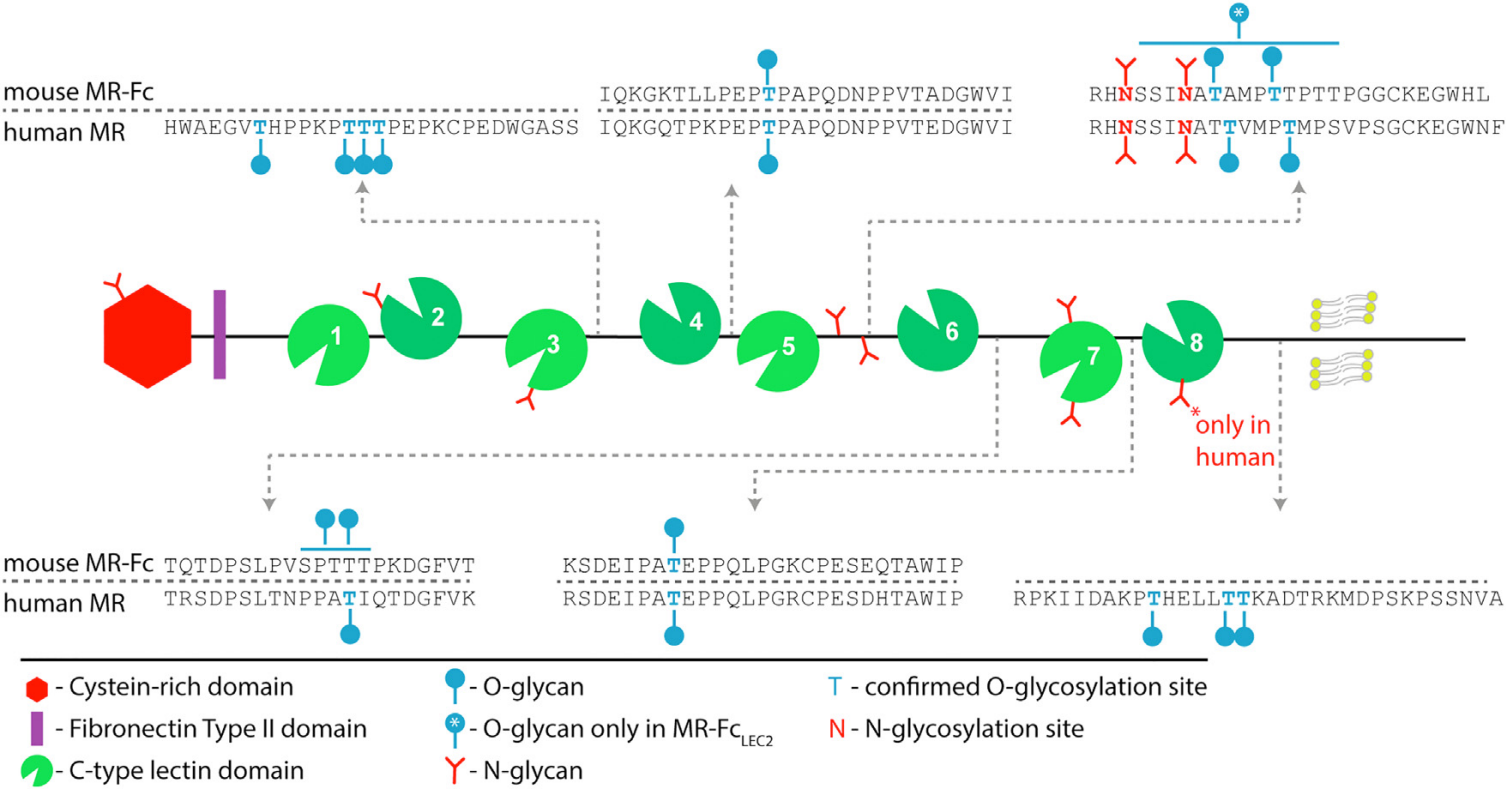
**MR O-glycosylation site identification.** A schematic representation of the MR illustrates all identified O-glycosylation sites based on the murine CTLD4-7 in MR-Fc_HEK-WT_, MR-Fc_CHO-WT_, MR-Fc_Lec2_, MR-Fc_PIR-P3_, and the human full-length MR. The sequences of the CTLD linker regions in murine and human MR-Fc are shown. Identified O-glycosylation sites are mainly located in the linker regions of the CTLDs. Between CTLD6-7 and CTLD5-6, the exact position of total three O-glycosylation sites could not be identified and thus, regions are indicated. CTLD, C-type lectin domain; MR, mannose receptor.

The site-specific glycopeptide analysis of MR-Fc, containing the murine CTLD4-7, revealed the presence of O-glycosylation in the receptor portion. We further analyzed the full-length human full-length MR to evaluate the conservation of these O-glycosylation sites between human and mouse MR. Up to seven occupied O-glycosylation sites were identified in MR-Fc and up to 12 in the full-length human MR (Fig. 5; representative spectra of each site are provided in Figs. S8–S17). Interestingly, all the identified sites were located in the linker region between the individual CTLDs.

The linker region of CTLD3-4 (His627-Lys654) contains up to four occupied O-glycosylation sites at Thr633, Thr639, Thr640, and Thr641 in the full-length human MR. In both murine MR-Fc and human full-length MR, Thr790 was found to be O-glycosylated, located within the linker region CTLD4-5 (Ile779-Ile806) (Fig. 5). The region between CTLD5 and CTLD6 (Arg924-Phe951 - human, Arg924-Leu950 - murine) was the most complex one, since it also contains two N-glycosylation sites. The O-glycopeptides were analyzed after N-glycan removal, revealing Thr932 and Thr936 to be O-glycosylated in MR-Fc. Another site within the region of Ser927-Thr940 was found in MR-Fc_Lec2_ only. The same linker region in human full-length MR featured up to two occupied glycosylation sites at Thr933 and Thr937. The latter one corresponds to Thr936 in mouse MR-Fc. Between CTLD6 and CTLD7 (Thr1081-Lys1101 – human, Thr1080-Thr1100 - murine), two glycosylation sites were identified within the region Ser1089-Thr1093 in MR-Fc; the same region where Thr1093 was identified in the human full-length MR. Another conserved site at Thr1220 (murine)/Thr1221 (human) was found in the region between CTLD7 and CTLD8 (Arg1214-Pro1240 – human, Lys1213-Pro1239 - murine). The spacer between CTLD8 and the transmembrane domain of MR was present only in the human full-length MR and it contained up to three occupied O-glycosylation sites at Thr1366, Thr1371, and Thr1372 (Fig. 5).

In terms of the O-glycan structural features, all CHO MR-Fc carries core 1 O-glycans or the Tn antigen, and MR-Fc_HEK-WT_ additionally carries core 2 O-glycans. The relative O-glycan distributions of MR-Fc_CHO-WT_ and MR-Fc_PIR-P3_ are highly similar, with HexNAc_1_Hex_1_NeuAc_1_ as the main structure, while MR-Fc_Lec2_ contains the same glycans without sialic acid (Fig. S19). In contrast, MR-Fc_HEK-WT_ contains nearly equal amounts of both sialylated and neutral O-glycans (Fig. S18).

## Discussion

Here, we identified the role of MR glycosylation in regulating the glycan-binding ability of its CTLDs. A combination of glyco-enzyme treatments and site-specific glycosylation site characterization by LC-MS revealed the importance of masking terminal, nonreducing GlcNAc residues to maintain MR-Fc CTLD glycan *trans*-binding (Fig. 3*D*). The MR-Fc expressed in either Lec2 or PIR-P3 CHO cells was inactive, but the activity was restored after N-glycan removal, demonstrating the involvement of N-glycosylation in mannose binding. The active receptor loses its ability to bind mannose when being treated with neuraminidase and galactosidase, which results in N-glycans with terminal GlcNAc. Additionally, the expression of MR-Fc in Lec2 CHO cells was characterized by partially under-galactosylated N-glycan antennae, exposing terminal GlcNAc, and resulting in an inactive MR-Fc. In return, the mannose-binding ability can be regained by masking terminal GlcNAc on N-glycan antennae with galactose or by complete N-glycan removal of the inactive receptor. Mannose-terminating N-glycans are a feature of MR-Fc expressed in PIR-P3 CHO cells, which showed no glycan-binding ability. Our results suggest that terminal mannose and GlcNAc present self-ligands to the MR CTLDs, potentially resulting in self-glycan binding or oligomerization and thus, inability for *trans*-glycan binding.

Our results are overall in agreement with a study by Su *et al.*, (16) although our additional experiments lead to different conclusions. This prior study investigated the glycan-binding ability of MR expressed in CHO Lec2 (defect in CMP–sialic acid transport) and CHO Lec1 (mainly Man_5_GlcNAc_2_ N-glycans) cells compared to WT CHO cells (16). In agreement with our results, it was found that Lec1 and Lec2 CHO cells expressing surface-bound MR were unable to bind mannose ligands. In addition, the ability of the MR in those cells to endocytose was hardly affected, and binding of sulfated galactose through the cysteine-rich domain was reduced but not abolished. Accordingly, the soluble-secreted full-length receptor expressed in Lec1 and Lec2 CHO cells also did not bind mannose but could bind sulfated galactose. Released N-glycan analysis of the full-length MR expressed in Lec2 CHO cells revealed Hex_5_HexNAc_4_Fuc_1_ as the major N-glycan. These results led the authors to conclude that sialylation is the critical glycosylation feature that regulates the activity of MR (16).

However, in our study, we used additional approaches employing exoglycosidase treatment, such as neuraminidase and galactosidase, as well as complete N-glycan removal by PNGase F on the recombinant full-length MR and MR-Fc, to identify MR regulation by unmasking or masking of terminal GlcNAc-containing N-glycan antennae. We characterized the site-specific MR-Fc glycosylation and found that two out of four MR-Fc N-glycosylation sites carry mainly Hex_3_HexNAc_4_Fuc_1_, a biantennary N-glycan with unmasked terminal GlcNAc, while the other two sites carry fully galactosylated N-glycans. The presence of Hex_5_HexNAc_4_Fuc_1_ as the major MR N-glycan in the previous study by Su *et al.* (16) is most likely reflecting an average of the overall full-length MR glycosylation. Our results clearly highlight the importance of performing site-specific glycopeptide analyses as a complementary approach for the analysis of released N-glycans.

In further support of our findings is the delayed MR activation during biosynthesis throughout the endoplasmic reticulum (ER)-Golgi compartment in monocyte-derived macrophages (22). MR activation was correlated with the development of Endo H resistance, because of N-glycan maturation throughout the ER and Golgi compartments. Thus, MR activation must take place after leaving the ER and is independent of O-glycosylation and complete maturation in the *trans*-Golgi, where sialylation is predicted to occur. In addition, treatment with brefeldin A, which inhibits transport to the *trans*-Golgi, needed for glycan maturation, resulted in reduced but not abolished MR activity. Consequently, it was concluded that activation of the MR takes place in the early Golgi (22), which correlates with the addition of galactose to hybrid and complex N-glycans.

GlcNAc has been reported as a lower affinity ligand of the MR CTLDs (8, 9, 11, 12), since it contains a pair of equatorial 3- and 4-OH groups in the same stereochemistry as mannose (23). While recombinantly expressed CTLD1-3 or CTLD6-8 do not bind GlcNAc, the CTLD4-5 binds to GlcNAc and also to mannose (8). Recently, we investigated endogenous MR-Fc ligands using glycan microarrays, and no binding was observed to GlcNAc-terminating structures representing glycans or glycan fragments of the mammalian glycome. However, we also observed that the MR-Fc bound to GlcNAc_3_-AEAB on the *S. mansoni* glycan microarray (13) as well as to high-density GlcNAc-agarose beads (Fig. S6), indicating that these low-affinity interactions are enhanced by multimeric or multivalent presentation.

MR has been reported to aggregate in the presence of Ca^2+^, which can be reduced by treating the receptor with Endo H and/or adding α-methylmannose (24). In that report, sedimentation velocity experiments suggest that if the receptor binds another glycan on the receptor, it might be most likely due to the binding of another receptor molecule (oligomerization) but not due to internal receptor binding and bending of the structure. Similarly, in the presence of Ca^2+^, oligomerization of the receptor was detected, which is not the case in the presence of EDTA and added mannose using gel filtration chromatography (25). It can be speculated that part of the receptor in the previous study carried oligomannose N-glycans, resulting in receptor oligomerization. Also, the presence of EDTA or mannose did not change the migration of MR in gel filtration, whereas migration was changed upon change in pH, suggesting a conformational change (25). All of these studies indicate that the presentation of receptor self-ligands results in dimerization or oligomerization rather than self-binding; however, this needs to be further investigated.

Differential amounts of monomeric and dimeric MR have been extracted from a rat liver and lung (26). While the monomeric form is specific to mannose binding, the dimeric form was needed for stable receptor binding with sulfated GalNAc *via* the cysteine-rich domain (26). It can be speculated that the presentation of receptor self-ligands increases dimerization, which leads to differential glycan recognition and functional properties of the receptor in a tissue-specific manner.

We confirmed seven MR N-glycosylation sites that are conserved between humans and mice. In addition, seven O-glycosylation sites were identified in MR-Fc and 12 in the human full-length MR, all localized in the linker regions between the CTLDs. An early sequencing study of the human MR revealed the presence of O-glycosylation in the linker region between CTLD3-4, corresponding to position Thr639/Thr640/Thr641 (15). We also found up to four O-glycosylation sites in this linker region, confirming Thr639/Thr640/Thr641. Another O-glycosylation site previously reported was Thr1093 in the linker region between CTLD6 and CTLD7, which was also confirmed in our mouse and human versions by mass spectrometric analysis.

Hydrodynamic analyses in combination with proteolysis experiments revealed some further structural information about MR (24). The linker regions on both sites of CTLD3 and CTLD6 are flexible and exposed to proteases. In contrast, close contact was reported between CTLD1-2, CTLD4-5, and CTLD7-8 in proteolytic experiments (24). The tight link between CTLD4-5 has been also confirmed by a separate proteolysis study using CTLD4-7 (10). Interestingly, these results correlate with our O-glycosylation site identification. The linker in between tightly connected CTLDs carries either no identified O-glycosylation site (CTLD1-2) or a single conserved O-glycosylation site as found between CTLD4-5 (Thr790) and CTLD7-8 (Thr1220 murine/1221 human). The linker regions that were found to be more exposed to protease tend to carry more O-glycosylation sites.

Overall, a very rigid structure of the receptor was found in modeling studies in combination with experimental studies, which indicated a mainly extended structure of the receptor (24) and it was speculated that larger flexibility in some of the linker region might be favorable for adjusting to binding a larger variety of glycans. However, EM-based studies were used to elucidate the three-dimensional structure of the receptor, indicating that it can be present in the bend and extended conformation, which is pH-dependent (25, 27). Considering that the receptor is shuttling between the plasma membrane and endosomal compartment where the ligand is released, this conformational change could be a regulatory mechanism. In agreement with the previous study, the three-dimensional structure also revealed tightly packed CTLD4-5 and CTLD7-8 (25).

Our study revealed that the function of the MR CTLDs can be modulated by their own glycosylation. Differential MR glycosylation has been reported across a variety of tissues (17) and it can be speculated that this might be a regulatory mechanism to control the activity of MR CTLDs in a tissue- or cell-specific manner. Similarly, sialic acid–binding immunoglobulin-like lectins (Siglecs) bind sialylated self-glycans or glycans on neighboring cell surface proteins, which can inhibit *trans*-binding of other cells, and enzymatic desialylation/resialylation could regulate Siglec *trans*-binding (28). On the contrary, certain pathogens may use this regulatory switch of unmasking MR CTLD self-ligands on the receptor and thus, inactivating receptor *trans*-binding to their advantage to escape from the immune system. A variety of bacteria secrete enzymes, including β1,4-galactosidases, in order to hydrolase glycan epitopes, which can have immunomodulatory effects for pathogenesis (29, 30). Our data sheds new light on the structure–function relationship of MR glycosylation, and further studies will be needed to determine to what extent this switch is implemented in biological processes.

## Experimental procedures

### Materials

Human recombinant full-length MR with His-tag, expressed in mouse myeloma cell line NS0, was purchased from R&D systems, anti-human IgG Alexa-Fluor 488-conjugated from Jackson ImmunoResearch, and anti–penta-His Alexa-Fluor 488 conjugate from Qiagen. Biotinylated Concanavalin A, *R. communis* agglutinin-I, *Maackia amurensis* lectin-I, *Sambucus nigra* lectin, fluorescein Concanavalin A, fluorescein Wheat germ agglutinin, and Man-BSA were purchased from Vector Laboratories; neuraminidase and Protein A-agarose beads from Roche; O-glycosidase, PNGase F, α-galactosidase, β1,3-galactosidase, and β1,4-galactosidase from New England Biolabs; sequencing grade trypsin from Promega; Glucitol-polyacrylamide (PAA), Man_3_-PAA, and Glucitol-PAA biotin from Lectinity; GlcNAc and GlcNAc-BSA from Carbosynth. All other chemicals were purchased from Sigma-Aldrich if not indicated differently.

### MR-Fc expression and purification

HEK293 WT, CHO WT, CHO Lec2, and CHO PIR-P3 cells were transfected with MR-Fc DNA, comprising the murine CTLD4-7, fused to the Fc-portion of human IgG (31) (kind gift from L. Martinez-Pomares). CHO PIR-P3 cells were kindly provided by Dr Mark Lehrman (UT Southwestern Medical Center) (21) and all other lines were obtained from ATCC. Transfection was performed with Lipofectamine LTX with Plus Reagent (Thermo Fisher Scientific), according to the manufacturer’s guidelines. The cells were incubated with Lipofectamine complex at 37 °C in a CO_2_ incubator for 24 h, followed by replacing the medium. HEK293 WT cells were transfected with MR-Fc and were cultured in RPMI culture media with 10% heat-inactivated fetal bovine serum (HI FBS), 1% penicillin-streptomycin (PS), and 10 mM Hepes containing nonessential amino acids; CHO WT cells were grown in MEM alpha (no nucleosides) Gibco 12561049, with 10% fetal calf serum, and PS (100 IU/100 μg/ml) finally added. CHO PIR-P3 cells were cultured in RPMI with 10% HI FBS and 1% PS-glutamine; CHO Lec2 cells were cultured in αMEM with ribonucleosides and deoxyribonucleosides in 10% HI FBS. The supernatant, containing the MR-Fc, was collected after 9 days. MR-Fc was purified from cell culture supernatant using Protein A-agarose beads. The supernatant was incubated with the beads at 4 °C on a rotator overnight, followed by three washes with PBS and MR-Fc elution using 0.1 M Glycine-HCl, pH 2.7 and immediate neutralization with 1 M Tris–HCl, pH 8.7. MR-Fc was stored at −20 °C until further use.

### MR-Fc enzymatic treatment prior microarray analysis

Prior to microarray analysis, 10 μl MR-Fc (2 μg/μl MR-Fc_HEK_, 0.13 μg/μl MR-Fc_CHO-WT_) was mixed with 15 μl PBS and 2 μl neuraminidase was added, followed by incubation at 37 °C overnight. Control samples without neuraminidase were included. Similarly, for PNGase F treatment, 10 μl MR-Fc (2 μg/μl MR-Fc_HEK-WT_, 0.13 μg/μl MR-Fc_CHO-WT_, 0.35 μg/μl MR-Fc_CHO-Lec2_, 0.18 μg/μl MR-Fc_CHO-PIRP3_) was mixed with 15 μl PBS and 1 μl PNGase F was added, followed by incubation at 37 °C overnight. MR-Fc and human full-length MR were also subjected to neuraminidase and subsequent galactosidase treatment. Human full-length MR (10 μl of 50 ng/μl) was treated with 2 μl β1-4 galactosidase and 2 μl α-galactosidase, according to the manufacturer’s instruction, with the addition of 1 μl neuraminidase and incubated at 37 °C overnight. Similarly, 6 μl MR-Fc_HEK_ (0.85 μg/μl) or 3 μl MR-Fc_CHO-WT_ (0.14 μg/μl) were treated with 2 μl β1,4-galactosidase or β1,3-galactosidase, according to the manufacturer’s instruction, with the addition of 1 μl neuraminidase and incubation at 37 °C overnight.

To study the effect of MR-Fc regalactosylation, a larger amount of MR-Fc_HEK-WT_ was treated accordingly with neuraminidase and β1,4-galactosidase. To remove excess enzymes, degal-MR-Fc_HEK_ was purified using Protein A agarose beads as described previously. MR-Fc_HEK-WT_ and degal-MR-Fc_HEK-WT_ in PBS (for each 20 μl of 0.1 μg/μl) were de–N-glycosylated with 1 μl PNGase F and incubated at 37 °C overnight. In addition, degal-MR-Fc_HEK-WT_ regalactosylation was performed by adding 3 μl of β-1,4-Galactosyltransferase 1 (β4GalT1/0.5 μg/μl, from Dr Kelley Moremen, glycoenzymes.ccrc.uga.edu) to 30 μl of 0.07 μg/μl degal-MR-Fc_HEK-WT_ in 20 mM Tris–HCl, pH7.4, 150 mM NaCl, 2 mM CaCl_2_, 20 mM MgCl_2_, 20 mM MnCl_2_, and 1 mM UDP-Gal (Chemily Glyoscience), followed by incubation at 37 °C overnight.

### Microarray printing Man-BSA array

We developed a customized glycan microarray to test MR-Fc glycan-binding activity. Different presentations of mannose were used, such as Man3-BSA, Man3-PAA, Man-BSA and the corresponding controls Glu-PAA, biotinylated Glu-PAA, GlcNAc-BSA, BSA, MR-Fc_HEK-WT_, and PBS. All probes were printed onto Nexterion Slide H NHS Slides (Schott AG), using the sciFLEXARRAYER S11 (Scienion US, Inc) equipped with piezo-dispensing capillary nozzles with a modified Type 3 coating (PDC 70 Type 3) as sold by the manufacturer. Careful adjustments were made to the voltage and pulse parameters so as to dispense 330 ± 10 pl per spot, and four spots per probe were printed per subarray on the slide. In total, there were 16 subarrays per slide. In some cases, additional probes may have been printed which were irrelevant to the current study so as to minimize costs. After the print, the slides were incubated overnight at 70% relative humidity and room temperature (RT). The next day, any unreacted surface of the slides was blocked by placing the slides in a solution of 50 mM ethanolamine in borate buffer (100 mM sodium tetraborate buffer pH 8.5) for 1 h. Slides were then removed from this solution one-by-one, dip-washed 10 times in PBS containing 0.05% Tween-20 and then ten times in water, following which they were dried using a centrifuge. Slides were stored at −20 °C prior to use. Note that, since the probes were very different in nature (*e.g.*, protein linker *versus* polyacrylamide linker with variations in the stoichiometry of conjugation to the glycan), their concentrations were kept proportional to that provided by the manufacturer and diluted with buffer to one or more dilutions so that the majority of the buffer was PBS. Final concentrations of all probes are listed in Table S3. As a control for slide printing, appropriate lectin controls were assayed and analyzed.

### Microarray screening

Glycan microarrays were screened using a standard protocol as published elsewhere (32). Briefly, MR-Fc binding was performed in tris salts metals buffer (TSM) binding buffer [TSM (20 mM Tris–HCl, pH7.4, 150 mM NaCl, 2 mM CaCl_2_, and 2 mM MgCl_2_) + 0.05% Tween 20 + 1% BSA] for 1 h at RT on a shaker, followed by four washes with TSM + 0.05% Tween 20 and incubation with a secondary antibody (goat anti-human IgG Alexa-Fluor 488-conjugated, 5 μg/ml) in TSM-binding buffer for 1 h at RT on a shaker. The slides were washed four times with TSM + 0.05% Tween 20, followed by TSM and H_2_O. As a control, the binding was performed in the presence of 5 mM EDTA instead of CaCl_2_. The arrays were scanned using a GenePix 4300A scanner (Molecular Devices) at 488 nm, and data readout was performed using the GenePix Pro software package.

The following glycan microarrays were screened for binding under the same conditions as mentioned above: MR-Fc binding was analyzed on the CFG glycan array (32) (version 5.3) at 16 μg/ml MR-Fc_HEK-WT_, 14 μg/ml MR-Fc_CHO-WT_, 24 μg/ml MR-Fc_Lec2_, 32 μg/ml MR-Fc_PIR-P3_; oligomannose array (33) at 10 μg/ml MR-Fc_HEK-WT_ and 15 μg/ml for all CHO-based MR-Fc; on the Man-BSA array at 1 μg/ml. The human full-length MR was analyzed for binding on the Man-BSA array at 16 μg/ml with an anti-penta-His Alexa-Fluor 488 conjugate at 5 μg/ml. Details about the preparation of these arrays can be found in the corresponding references.

### MR-Fc enzymatic treatment before mass spectrometry analysis

For glycopeptide analysis, 2 μg MR-Fc from CHO cells, 8 μg MR-Fc_HEK-WT_, and 1 μg human full-length MR were treated with PNGase F, or mock treated, according to the manufacturer’s instructions. Alternatively, MR-Fc was treated with O-glycosidase according to the manufacturer’s instruction, including the addition of 2 μl neuraminidase. A portion of the human full-length MR PNGase F–treated samples was also incubated with 1 μl neuraminidase and further incubation at 37 °C overnight. The samples were subjected to SDS-PAGE and subsequent in-gel trypsin digestion as described elsewhere (34). Protein bands were digested with 20 μl of 0.0025 μg/μl sequencing grade trypsin in 25 mM ammonium bicarbonate overnight at 37 °C. The next day, the supernatant was collected and 50 μl of 50% acetonitrile was added and incubated for 10 min on a shaker. Both supernatants were combined and dried down in a speed vac concentrator. The samples were stored at −20 °C until further use.

### LC-MS glycopeptide analysis and data analysis

LC-MS was performed on an Ultimate 3000 nano LC coupled to an Orbitrap Fusion Lumos mass spectrometer (both Thermo Fisher Scientific), as described elsewhere (35). MR-Fc samples were diluted in 0.1% formic acid (FA) in H_2_O and loaded onto a C18 precolumn (C18 PepMap 100, 300 μm × 5 mm, 5 μm, 100 Å, Thermo Fisher Scientific) with 15 μl/min solvent A (0.1% FA in H_2_O) for 3 min and then separated on a C18 analytical column (PicoFrit 75 μm ID × 150 mm, 3 μm, New Objective) using a linear gradient of 2% to 45% solvent B (80% acetonitrile, 0.1% FA) over 29 min, followed by 45% to 90% B over 5 min at 400 nl/min. The mass spectrometer was operated under the following conditions: the ion source parameters were 1750 to 1850 V spray voltage and 200 °C ion transfer tube temperature. MS scans were performed in the orbitrap at a resolution of 60,000 within a scan range of *m/z* 400 to *m/z* 1600, a RF lens of 30%, and AGC target of 1e5 for a maximum injection time of 50 ms. The top five precursors were selected for MS^2^ in a data-dependent manner, within a mass range of *m/z* 400 to *m/z* 1600, a minimum intensity threshold of 5e4, and an isolation width of 2 *m/z*. Higher-energy collisional dissociation is performed in stepped collision energy mode of 25% or 28% or 30% (±5%) and detected in the orbitrap with a resolution of 30,000 with the first mass at *m/z* 120, an AGC target of 1e5, and a maximum injection of 200 ms. EThcD was performed in a product ion-dependent manner (*m/z* 204.0867) with 20% supplemental activation energy and detected in the orbitrap with a resolution of 30,000 with the first mass at *m/z* 120, an AGC target of 3e5, and a maximum injection of 250 ms. Detailed LC-MS settings for the analysis of the human full-length MR is provided in the supplementary information.

Glycopeptide identification was performed using Byonic version 3.5 (Protein Metrics Inc; https://proteinmetrics.com/byonic/). Trypsin was set as protease with a maximum of two missed cleavage sites, the precursor and fragment mass tolerance was set to 10 ppm. The glycan database was “N-glycan 309 mammalian” and “O-glycan 9 common”. The following modifications were allowed: carbamidomethyl (Cys; fixed), oxidation (Met; variable common 1), pyroglutamine on N-term (Gln, variable, rare 1), ammonia-loss N-term (Cys; variable rare 1), acetylation N-term (variable rare 1), deamidation (Asn, variable common 1), and formylation N-term (variable rare 1). Glycopeptides with a score above 250 were selected and further manually inspected.

Relative quantitation of all glycopeptides was performed in an automated manner as described previously (36). The glycopeptide reference list contained all glycopeptides that were identified based on MS^2^ fragmentation but also lower abundant glycopeptides based on their exact mass, corresponding retention time, isotopic pattern, and biosynthetic-related glycan composition. Relative intensities were determined based on triplicate analysis, and standard deviation was calculated.

## Data availability

The MS data have been deposited to the ProteomeXchange Consortium (http://proteomecentral.proteomexchange.org) *via* the PRIDE (37) partner repository with the dataset identifier PXD034781. The glycan microarray data will be available at the National Center for Functional Glycomics website, https://ncfg.hms.harvard.edu/ncfg-data/microarray-data.

Any remaining data is either presented in the article or freely available upon request to: Dr Richard D. Cummings, rcummin1@bidmc.harvard.edu.

## Supporting information

This article contains supporting information (35, 36).

## Conflict of interest

K. S. is an employee of AstraZeneca, after conduction of this research but prior to article submission.

## Supporting information

Supplemental Tables

Supplementary Experimental Procedures, Supplementary Table Legends, and Supplemental Figures S1-S19
