# Supplementary material for: N-glycosylation of mannose receptor (CD206) regulates glycan binding by C-type lectin domains
Source: J Biol Chem. 2022 Oct 13;298(12):102591. doi: 10.1016/j.jbc.2022.102591 (PMC9672410; doi:10.1016/j.jbc.2022.102591)
Supplement: Supplementary Experimental Procedures, Supplementary Table Legends, and Supplemental Figures S1-S19 [file mmc2.pdf]

Supplementary Information

N-glycosylation of Mannose receptor (CD206) regulates glycan binding by C-type lectin domains

Kathrin Stavenhagen[1,2], Akul Y. Mehta[1], Lisa Laan[2], Chao Gao[1], Jamie Heimburg-Molinaro[1], Irma van Die[2], Richard D. Cummings[1]

[1] Department of Surgery, Beth Israel Deaconess Medical Center, Harvard Medical School, Boston, MA, USA
[2] Department of Molecular Cell Biology and Immunology, Amsterdam UMC (VU Medical Center), Amsterdam, The Netherlands

Supplementary experimental procedures

Lectin blots to confirm enzymatic treatment

MR-Fc (0.5 - 0.65µg per lane) was subjected to SDS-PAGE and transferred onto a nitrocellulose membrane using the TransBlot Turbo system (BioRad). The membrane was blocked with 5% BSA in TBST (Tris-buffered saline with 0.05% Tween-20) for 1h and incubated with biotinylated SNA (2 µg/mL), MAL-I (2 µg/mL), ConA (0.1 µg/mL), or RCA-I (0.5 µg/mL) (Vector Laboratories) in 1% BSA in TBST overnight, followed by three washes in TBST for 10 min and incubation with streptavidin-HRP (Vector Laboratories) for 1h. As a control MR-Fc was probed with goat anti-human IgG-HRP (Jackson ImmunoResearch Laboratory Inc.) and incubated for 1h. After washing the membranes three times with TBST for 10 min the proteins were visualized using SuperSignal West Pico Plus Chemiluminescence Substrate (Thermo Scientific) and imaged.

MR-$Fc_{HEK\ WT}$ enrichment over GlcNAc-agarose beads

40 µL GlcNAc-agarose beads (Sigma) were washed three times with 500 µL of TBS, containing 5 mM $CaCl_2$. 40 µL of MR-$Fc_{HEK\text{-}WT}$ sample (9 µg each) in TSM binding buffer were incubated with the beads for 2 h on a shaker at RT. The beads were transferred to empty top tips (Glygen) and placed in an Eppendorf vial for separation by centrifugation. The flow through was collected and the beads were washed twice with 50 µL TBS, containing 5 mM $CaCl_{2,}$ followed by elution with 50 µL 100 mM α-methylmannose (Sigma), 100 mM GlcNAc (Sigma), 100 mM EDTA (Sigma) or 100 mM galactose (Sigma). All fractions were subjected to SDS-PAGE.

MR-$Fc_{HEK\ WT}$ enrichment over mannan-agarose beads

40 µL mannan-agarose beads (Sigma) were washed three times with 500 µL of TBS, containing 5 mM $CaCl_2$. 50 µL of desia-MR-$Fc_{HEK\text{-}WT}$ and MR-$FC_{HEK\text{-}WT}$ sample (8.5µg each) in TSM binding buffer were incubated with the beads for 2.5 h on a shaker at RT. The beads were transferred to empty top tips (Glygen) and placed in an Eppendorf vial for separation by centrifugation. The flow through was collected and the beads were washed twice with 50 µL TBS, containing 5 mM $CaCl_{2,}$ followed by elution with 40 µL laemmli buffer (Bio-Rad) and incubation at 70°C for 10 min. All fractions were subjected to SDS-PAGE.

LC-MS O-glycopeptide analysis of the full-length human rMMR

Liquid chromatography-mass spectrometry (LC-MS) was performed on an Ultimate 3000 nano LC coupled to an Orbitrap Fusion Lumos mass spectrometer (both Thermo Fisher) as described elsewhere [1].
Samples were dissolved in 14 µL of 0.1% formic acid (FA) in H2O and loaded onto a C18 precolumn (C18 PepMap 100, 300 µm x 5 mm, 5 µm, 100 Å, Thermo Scientific) with 15 µL/min solvent A (0.1% FA in H2O) for 3 min and separated on a C18 analytical column (PicoFrit 75 µm ID x 150 mm, 3 µm, New Objective) using a linear gradient of 2 % to 45 % solvent B (80% acetonitrile, 0.1% FA) over 66 min, followed by 45 % to 90 % B over 3 min at 400 nL/min. The mass spectrometer was operated under following conditions: The ion source parameters were 2100 V spray voltage and 200°C ion transfer tube temperature. MS scans were performed in the orbitrap at a resolution of 60,000 within a scan range of m/z 400 – m/z 1600, a RF lens of 30%, AGC target of 1e5 for a maximum injection time of 50 ms. The top 15 precursors were selected for MS2 in a data dependent manner, within a mass range of m/z 600 – *m/z* 1600 and a minimum intensity threshold of 5e4 and an isolation width of 1.2 *m/z*. HCD is performed with 30 % collision energy and detected in the orbitrap with a resolution of 30,000 with the first mass at *m/z* 120 an AGC target of 1e5 and a maximum injection of 150 ms.
EThcD was performed in a product ion-dependent manner (*m/z* 204.0867) with 25 % supplemental activation energy and detected in the orbitrap with a resolution of 30,000 with the first mass at *m/z* 120 an AGC target of 2e5 and a maximum injection of 250 ms.

Supplementary Tables- separate Excel file

Supplementary Table S1. CFG glycan microarray data.

Supplementary Table S2. Oligomannose glycan microarray data.

Supplementary Table S3. Mannose-BSA glycan microarray quality control.

Supplementary Table S4. Mannose-BSA glycan microarray data.

Supplementary Table S5. Identification of N-glycopeptides.

Supplementary Table S6. N-glycopeptide quantitation.

Supplementary Table S7. Mannose-BSA glycan microarray data with enzyme treatment.

Supplementary Table S8. Mannose-BSA glycan microarray data with inhibition.

Supplementary Figures

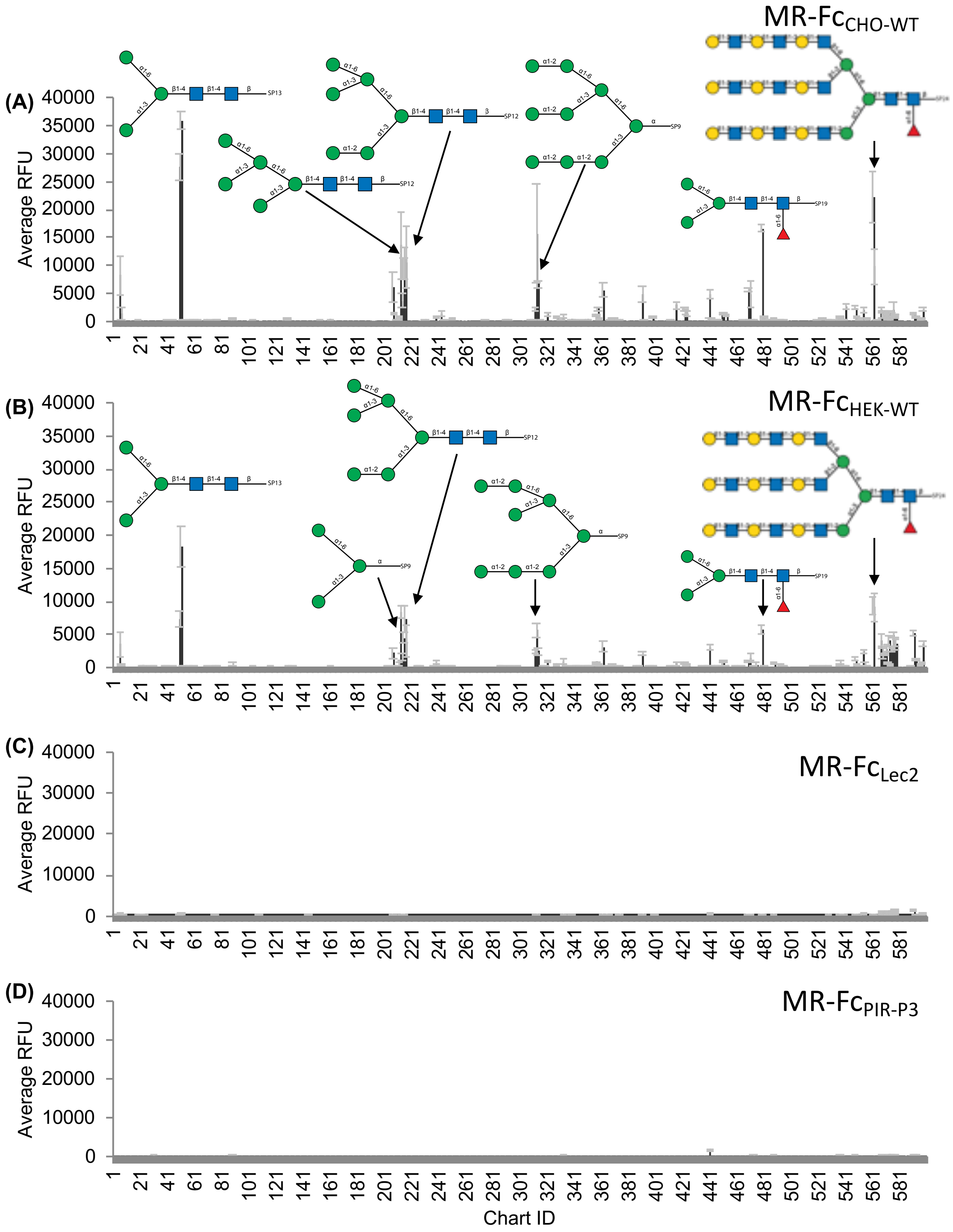

Figure S1: MR-Fc binding on CFG array. CFG array (version 5.3) was probed with **(A)** MR-Fc$_{CHO-WT}$ (14 µg/µL), **(B)** MR-Fc$_{HEK-WT}$ (16 µg/mL), **(C)** MR-Fc$_{Lec2}$ (24 µg/µL), **(D)** MR-Fc$_{PIR-P3}$ (32 µg/µL) and detection with goat anti-human IgG Alexa-Fluor 488-conjugated at 5 µg/ml. S+/- one standard deviation is shown.

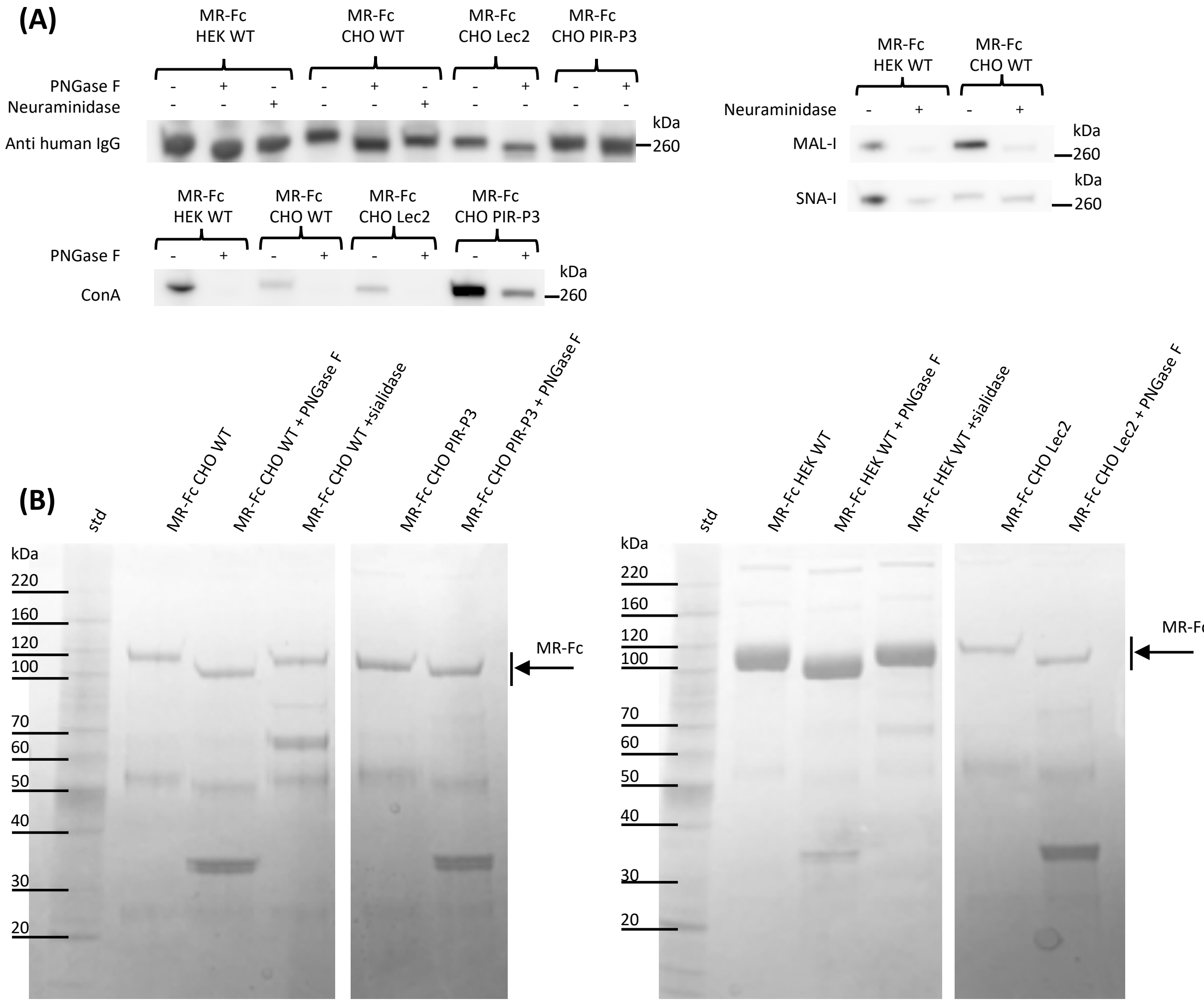

Figure S2: Evaluation of MR-Fc neuraminidase and PNGase F treatment. 2-8µg of MR-$Fc_{HEK\text{-}WT}$, MR-$Fc_{CHO\text{-}WT}$, MR-Fc_{Lec2} and MR-$Fc_{PIR\text{-}P3}$ were treated with PNGase F and neuraminidase as described in the method section. **(A)** Lectin blots confirming the enzymatic treatment of MR-Fc (0.65 µg per lane). **(B)** MR-Fc was subjected to SDS-PAGE under reducing conditions and subsequent Coomassie staining. Both the ConA lectin blot and the SDS-PAGE show a complete N-glycan removal by PNGase F of MR-$Fc_{HEK\text{-}WT}$, MR-$Fc_{CHO\text{-}WT}$ and MR-Fc_{LEc2} and a partial N-glycan release of MR-$Fc_{PIR\text{-}P3}$. Latter one might be due to self-binding of the MR-$Fc_{PIR\text{-}P3}$ to its N-glycans and thus partial inaccessibility of PNGase F. MAL-I and SNA lectin staining and SDS-PAGE confirm MR-Fc sialic acid removal.

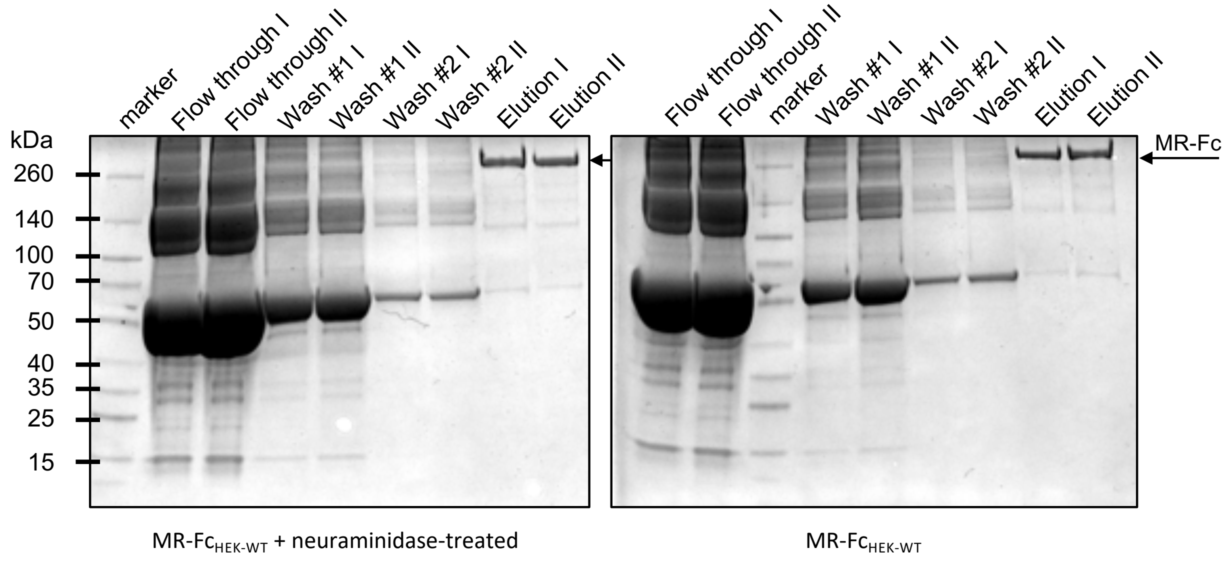

Figure S3: Sialic acid-independent MR-Fc enrichment over mannan-agarose beads. (A) Neuraminidase-treated MR-Fc$_{HEK-WT}$ and (B) mock-treated MR-Fc$_{HEK-WT}$ were enriched using mannan-agarose beads. The amount of MR-Fc bound to the beads is identical and thus, independent of MR-Fc sialylation. Experiments were performed in duplicates (I and II). 8.5 µg MR-Fc$_{HEK-WT}$ were desialylated as described in the method section.

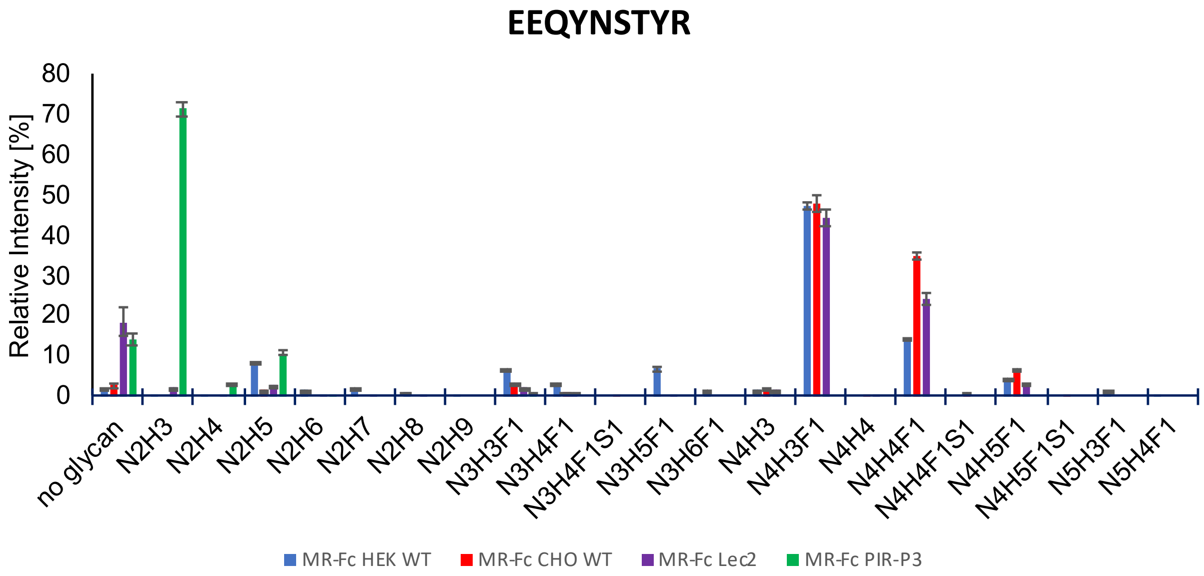

Figure S4: Relative N-glycopeptide quantitation of IgG Fc-part. All identified N-glycopeptides with the peptide portion EEQYNSTYR of the Fc-portion of MR-Fc (Suppl. Table S6) were used for relative quantitation as described in the method section. N – N-acetylglucosamine, H – Hexose, F – Fucose, S – N-acetylneuraminic acid. +/- one standard deviation is shown.

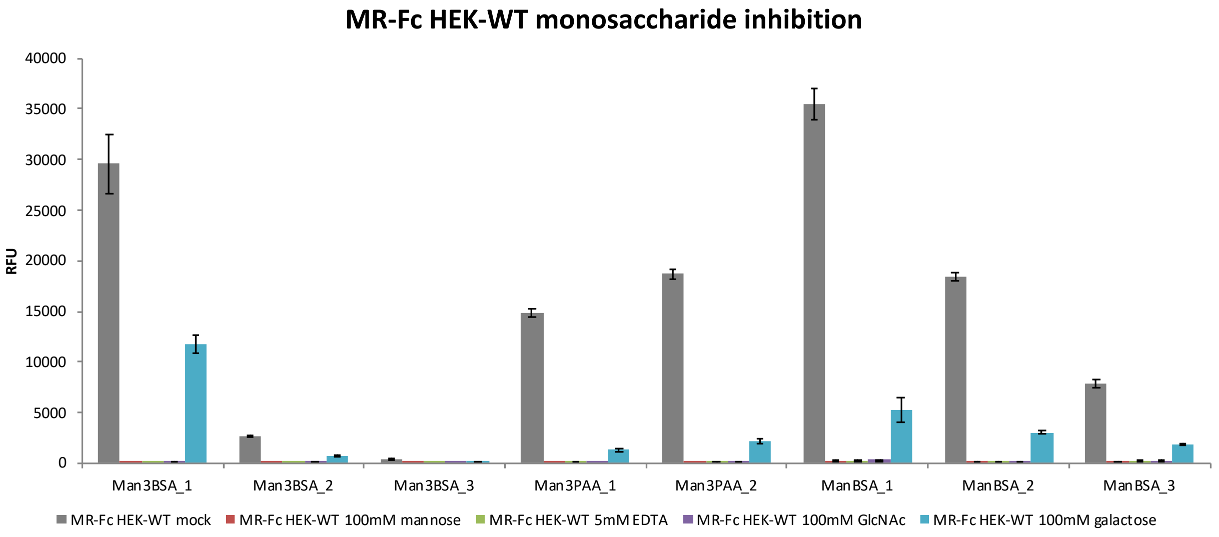

Figure S5: MR-Fc inhibition of mannose binding by GlcNAc. Man-BSA array was probed with $\text{MR-Fc}_{\text{HEK-WT}}$ (1ng/µL) in the presence of 100 mM α-methylmannose (Sigma), 100 mM GlcNAc (Sigma), 5 mM EDTA (Sigma) and 100 mM galactose (Sigma), preincubation for 30 min and detection with goat anti-human IgG Alexa-Fluor 488-conjugated at 5 µg/ml. Mannose, GlcNAc and EDTA completely inhibited MR-Fc binding to the Man-BSA array. In contrast, incubation with 100 mM galactose only partially reduced MR-Fc binding.

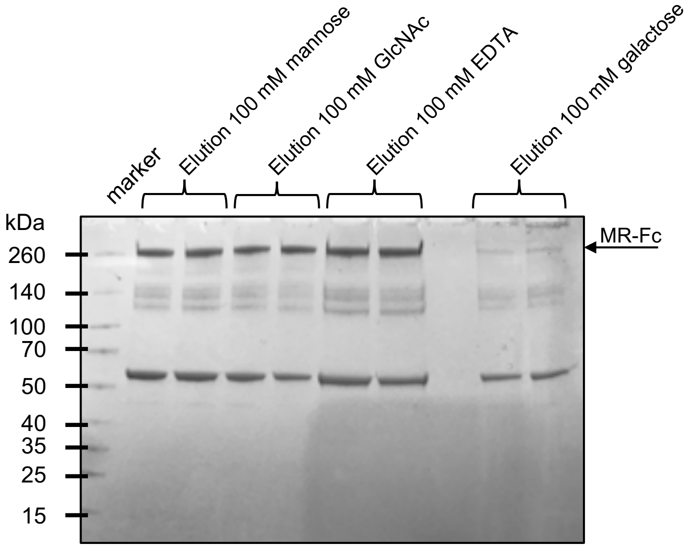

Figure S6: MR-Fc$_{HEK\ WT}$ enrichment over GlcNAc-agarose beads. MR-Fc$_{HEK\ WT}$ was enriched over GlcNAc-agarose beads and specifically eluted with 100 mM mannose, 100 mM GlcNAc, 100 mM EDTA, resulting in similar amounts of MR-Fc. The elution with 100 mM galactose only released a smaller portion of MR-Fc from the beads. Experiments were performed in duplicates.

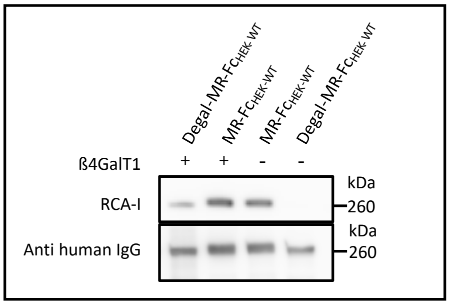

Figure S7: Regalactosylation of degalactosylated MR-Fc$_{HEK\text{-}WT}$ using β4GalT1. MR-Fc$_{HEK\text{-}WT}$ and degal-MR-Fc$_{HEK\text{-}WT}$ were enzymatically galactosylated using β4GalT1 and subjected to RCA-1 lectin blot to confirm partial regalactosylation of the previous degalactosylated MR-Fc. 0.5 µg per lane were loaded.

MR-Fc O-glycosylation site identification:

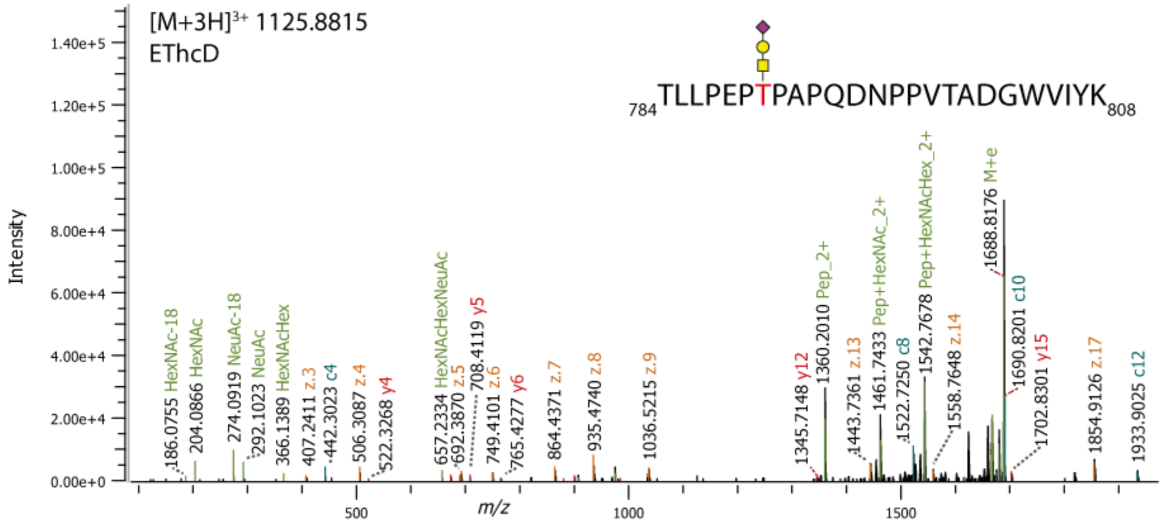

Figure S8: Identification of O-glycosylation site Thr790 in mouse MR. EThcD spectrum of the glycopeptide 784-TLLPEPTPAPQDNPPVTADGWVIYK-808 carrying the O-glycan $HexNAc_1Hex_1NeuAc_1$ at *m/z* 1125.8815 $[M+3H]^{3+}$ confirming O-glycosylation site Thr790.

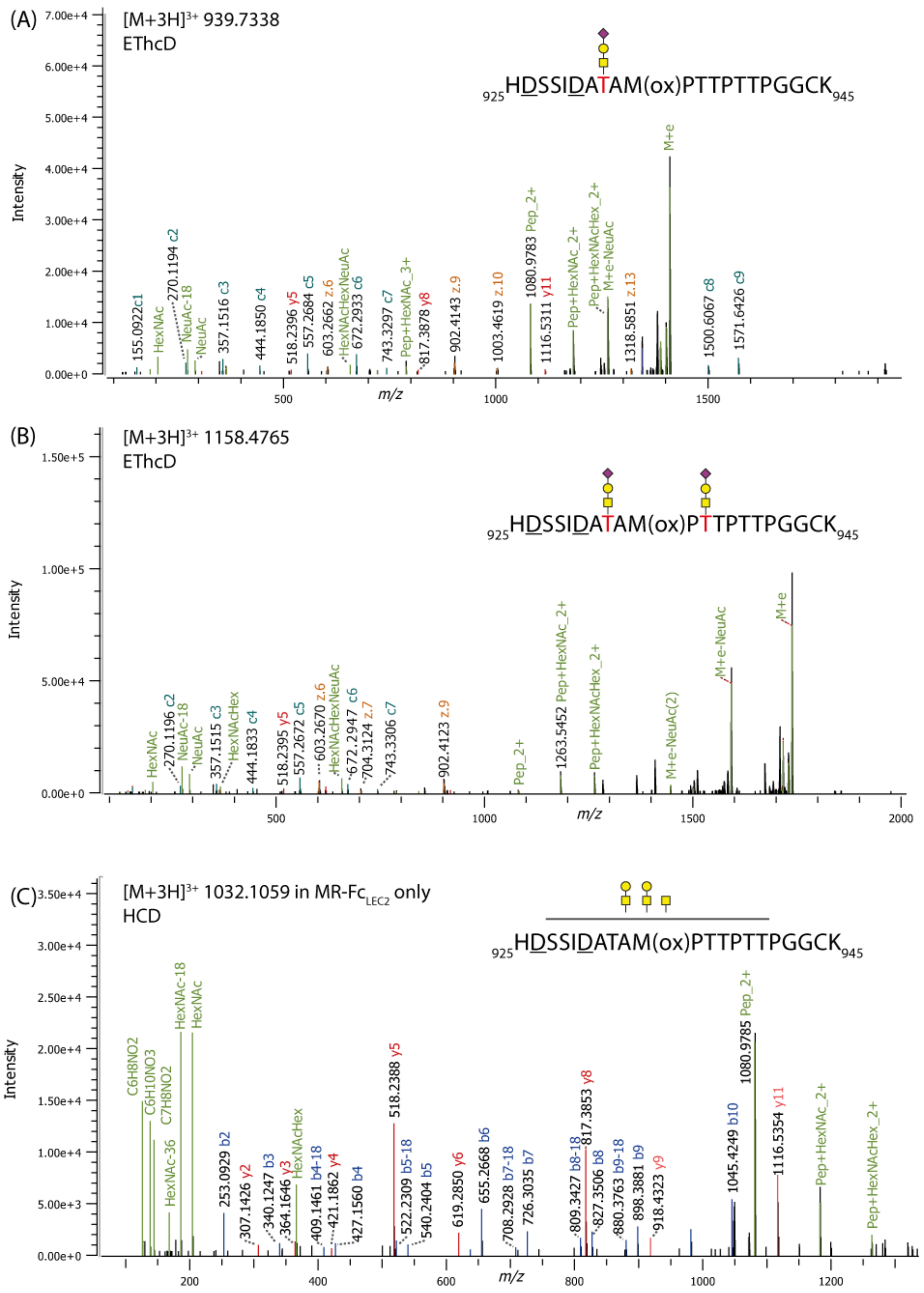

Figure S9: Identification of O-glycosylation site Thr932, Thr936 and an additional site between Ser927-Thr940 in mouse MR. **(A)** EThcD spectrum of the glycopeptide 925-HDSSIDATAM(ox)PTTPTTPGGCK-945 (D indicating deamidation by PNGase F treatment; (ox) indicating oxidation) carrying the O-glycan $HexNAc_1Hex_1NeuAc_1$ at *m/z* 939.7338 $[M+3H]^{3+}$ confirming O-glycosylation site Thr932. **(B)** EThcD spectrum of the glycopeptide 925-HDSSIDATAM(ox)PTTPTTPGGCK-945 (D indicating deamidation by PNGase F treatment; (ox) indicating oxidation) carrying the two O-glycans $HexNAc_1Hex_1NeuAc_1$ at *m/z* 1158.4765 $[M+3H]^{3+}$ confirming O-glycosylation sites Thr932 and Thr936. **(C)** HCD spectrum of the glycopeptide 925-HDSSIDATAM(ox)PTTPTTPGGCK-945 (D indicating deamidation by PNGase F treatment; (ox) indicating oxidation) carrying two O-glycans with the composition $HexNAc_1Hex_1$ and one with the composition $HexNAc_1$ at *m/z* 1032.1059 $[M+3H]^{3+}$ confirming three O-glycosylation sites in the peptide region Ser927-Thr940. The oxonium ion ratio suggests the presence of GalNAc only as a HexNAc, indicating the presence of three individual O-glycans attached to this peptide. These three O-glycosylation sites were only found in $MR\text{-}Fc_{Lec2}$.

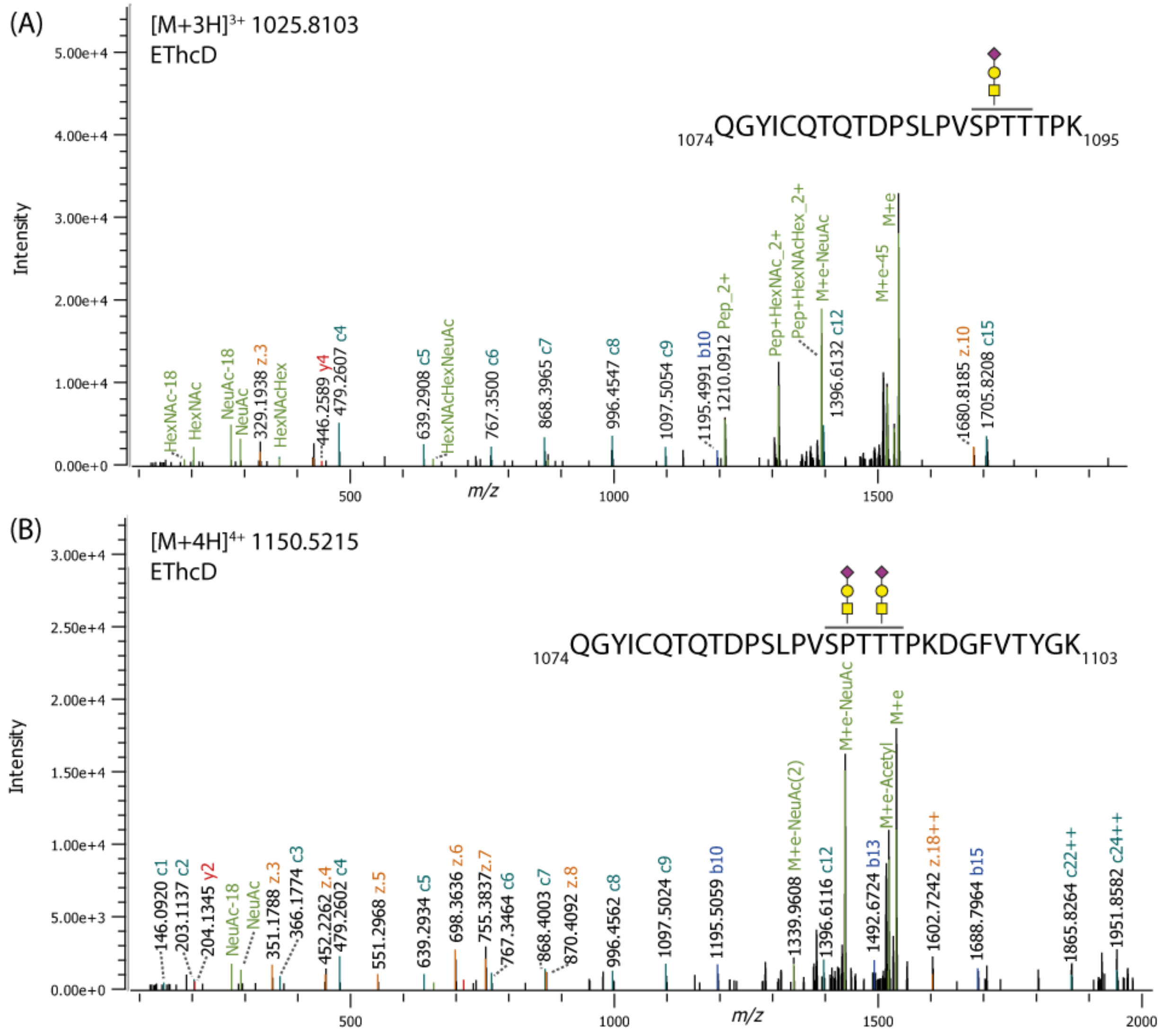

Figure S10: Identification of two O-glycosylation sites in the region Ser1089-1093 in mouse MR. **(A)** EThcD spectrum of the glycopeptide 1074-QGYICQTQTDPSSLPVSPTTTPK-1095 carrying the O-glycan $HexNAc_1Hex_1NeuAc_1$ at *m/z* 1025.8103 $[M+3H]^{3+}$ confirming one O-glycosylation site in the region of Ser1089-1093. **(B)** EThcD spectrum of the glycopeptide 1074-QGYICQTQTDPSSLPVSPTTTPKDGFVTYGK-1103 carrying two O-glycans with the composition $HexNAc_1Hex_1NeuAc_1$ at *m/z* 1050.5215 $[M+4H]^{4+}$ confirming two O-glycosylation sites in the region Ser1089-1093.

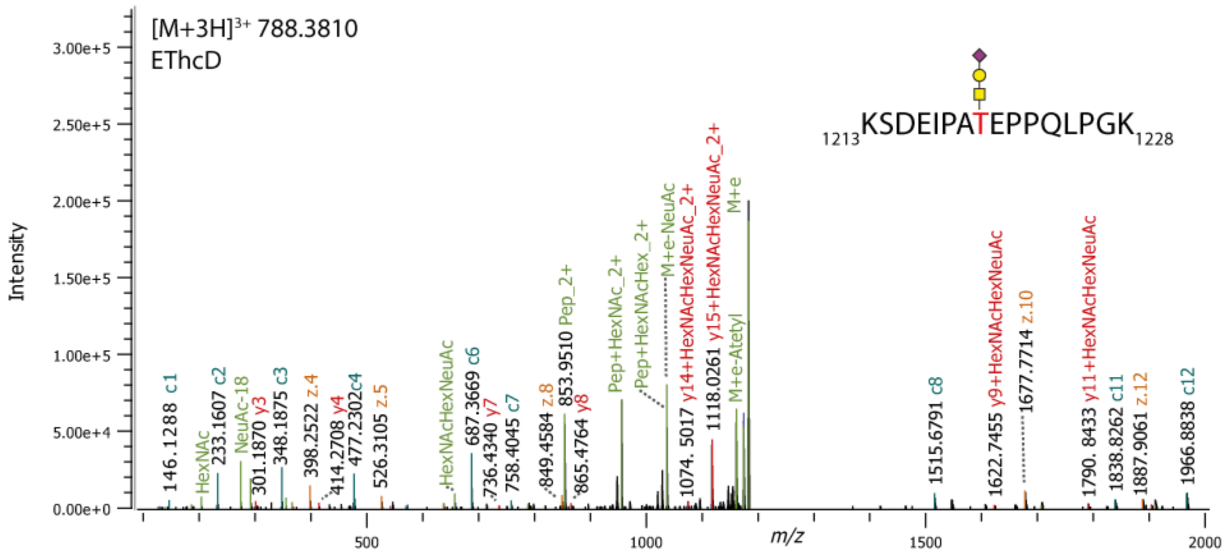

Figure S11: Identification of the O-glycosylation site Thr1220 in mouse MR. **(A)** EThcD spectrum of the glycopeptide 1213-KSDEIPATEPPQLPGK-1228 carrying the O-glycan $HexNAc_1Hex_1NeuAc_1$ at *m/z* 788.3810 $[M+3H]^{3+}$ confirming one O-glycosylation site in the region of Thr1220.

Human full-length mannose receptor O-glycosylation site identification

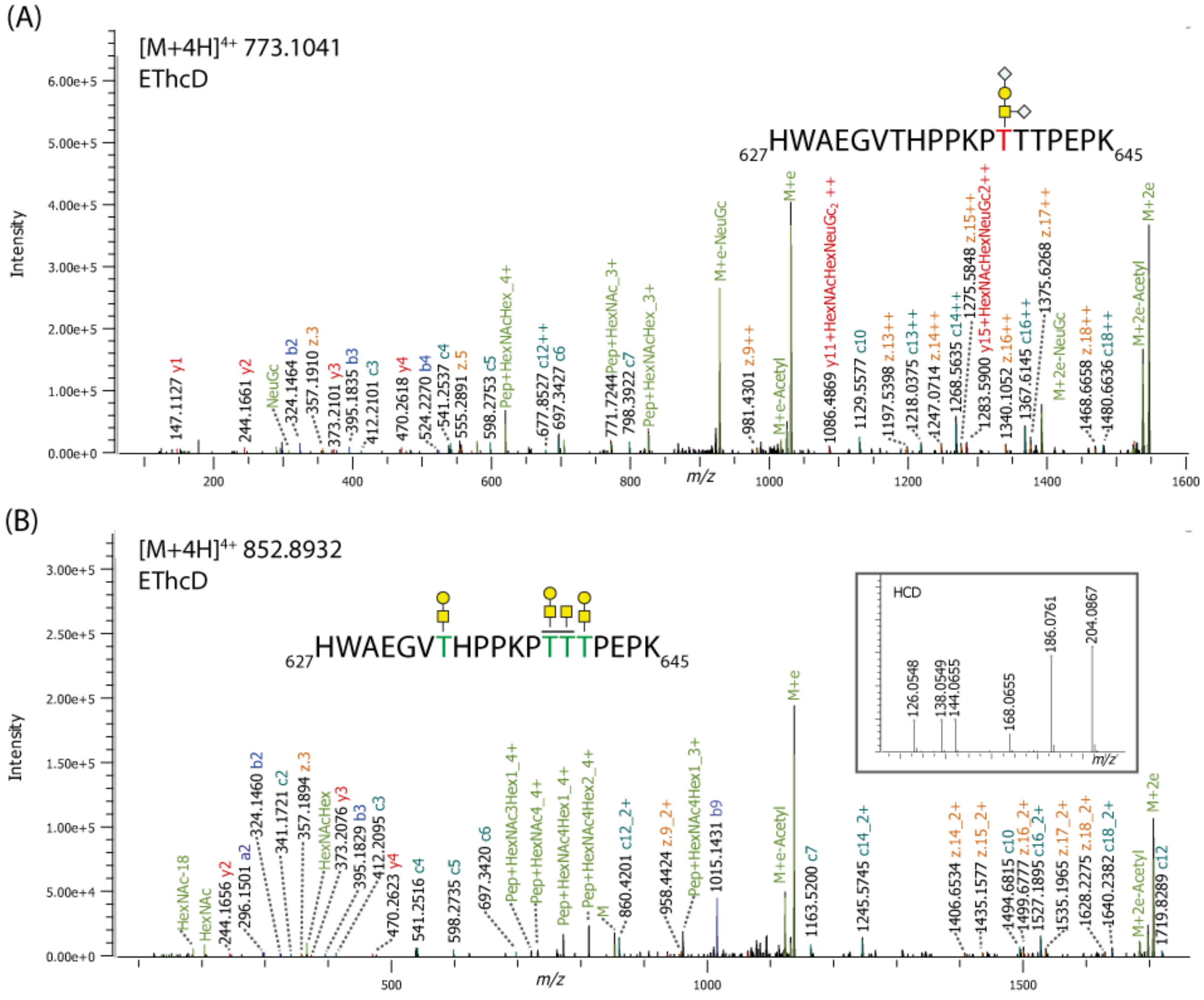

Figure S12: Identification of the O-glycosylation sites Thr633/Thr639/Thr640/Thr641 in human MR. **(A)** EThcD spectrum of the glycopeptide 627-HWAEGVTHPPKPTTTPEPK-645 carrying the O-glycans $HexNAc_1Hex_1NeuGc_2$ at *m/z* 773.1041 $[M+4H]^{4+}$ confirming one O-glycosylation site Thr639. (**B)** EThcD spectrum of the glycopeptide 627-HWAEGVTHPPKPTTTPEPK-645 carrying three O-glycans with the composition $HexNAc_1Hex_1$ and one with $HexNAc_1$ at *m/z* 852.8932 $[M+4H]^{4+}$ confirming the O-glycosylation sites Thr633/Thr639/Thr640/Thr641. Insert shows oxonium ion region in HCD spectrum.

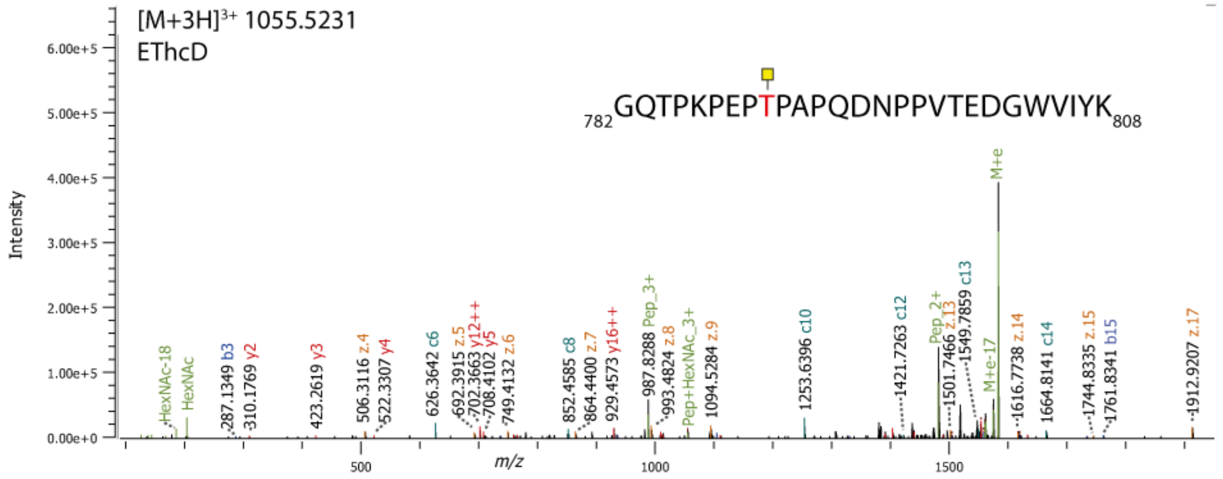

Figure S13: Identification of the O-glycosylation site Thr790 in human MR. EThcD spectrum of the glycopeptide 782-GQTPKPEPTPAPQDNPPVTEDGWVIYK-808 carrying the O-glycan $HexNAc_1$ at *m/z* 1055.5231 $[M+3H]^{3+}$ confirming one O-glycosylation site Thr790.

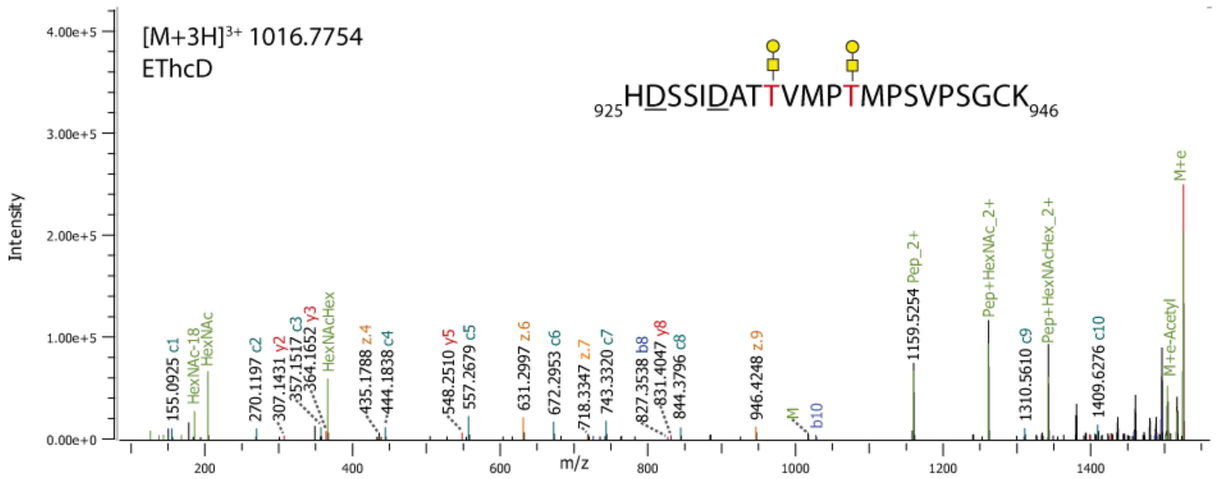

Figure S14: Identification of the O-glycosylation sites Thr933 and Thr937 in human MR. EThcD spectrum of the glycopeptide 925-HDSSIDATTVMPTMPSVPSGCK-946 (D indicated deamidation after PNGase F N-glycan release) carrying two O-glycans with the composition $HexNAc_1Hex_1$ at m/z 1016.7754 [M+3H]3+ confirming one O-glycosylation sites Thr933 and Thr937.

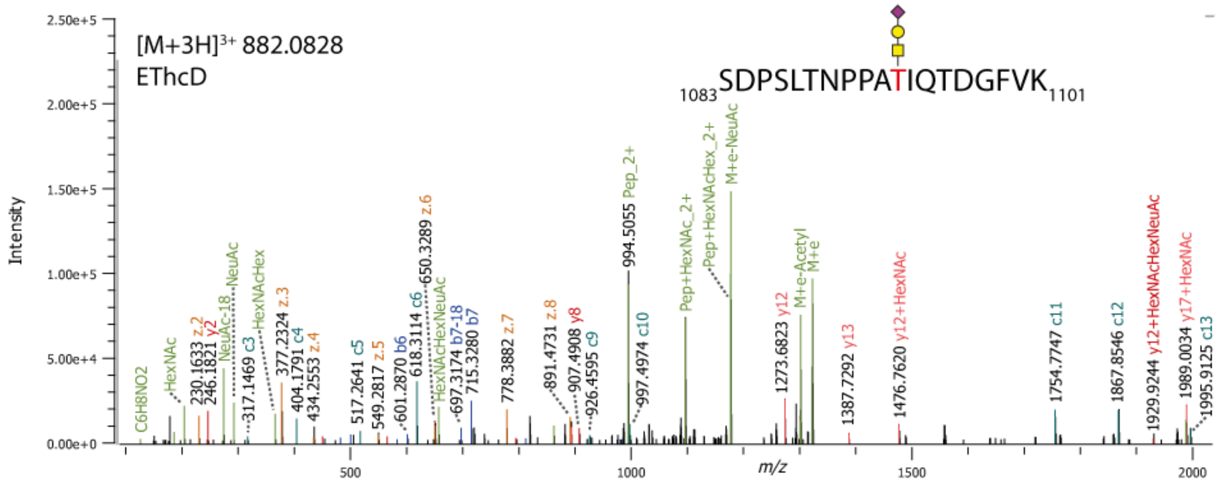

Figure S15: Identification of the O-glycosylation site Thr1093 in human MR. **(A)** EThcD spectrum of the glycopeptide 1083-SDPSLTNPPATIQTDGFVK-1101 carrying the O-glycan $HexNAc_1Hex_1NeuAc_1$ at *m/z* 882.0828 $[M+3H]^{3+}$ confirming one O-glycosylation site Thr1093.

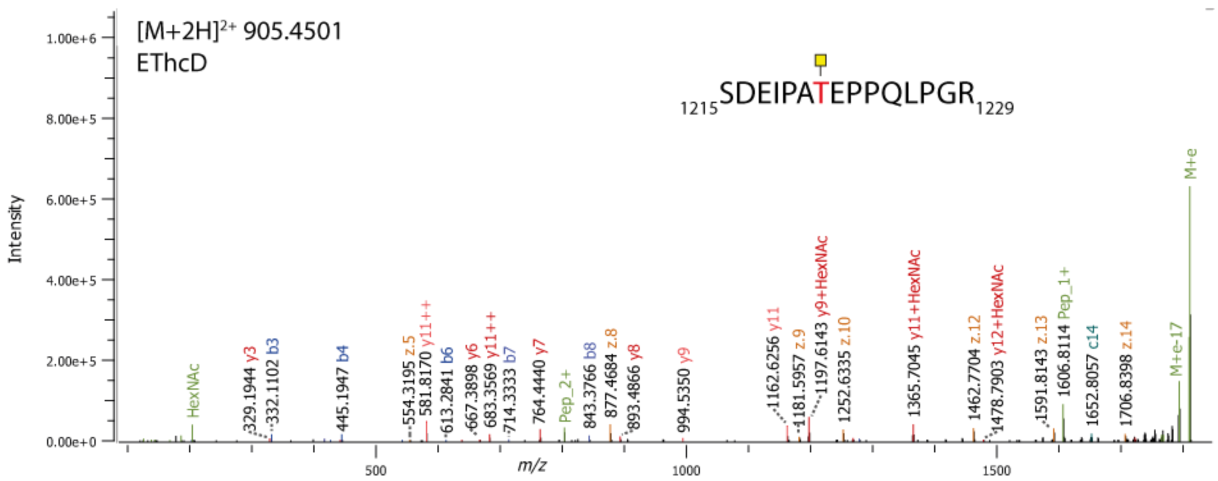

Figure S16: Identification of the O-glycosylation site Thr1221 in human MR. **(A)** EThcD spectrum of the glycopeptide 1215-SDEOPATEPPQLPGR-1229 carrying the O-glycan $HexNAc_1$ at *m/z* 905.4501 $[M+2H]^{2+}$ confirming one O-glycosylation site Thr1221.

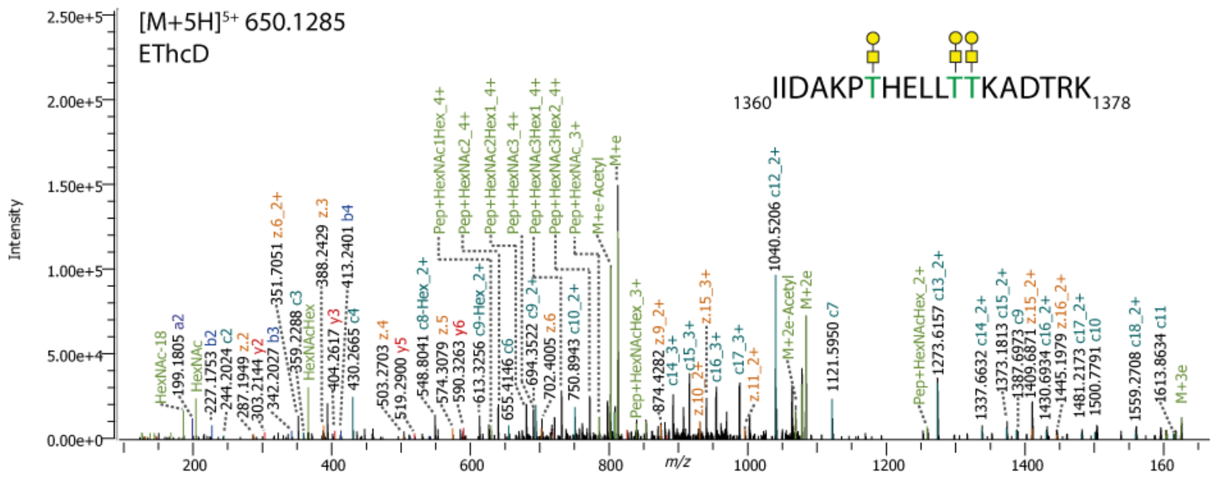

Figure S17: Identification of three O-glycosylation sites Thr1366, Thr1371, Thr1372 in human MR. **(A)** EThcD spectrum of the glycopeptide 1360-IIDAKPTHELLTTKADTR-1377 carrying three O-glycans with the composition $HexNAc_1Hex_1$ at *m/z* 650.1285 $[M+5H]^{5+}$ confirming three O-glycosylation sites Thr1366, Thr1371, Thr1372.

MR-Fc O-glycosylation site relative quantitation

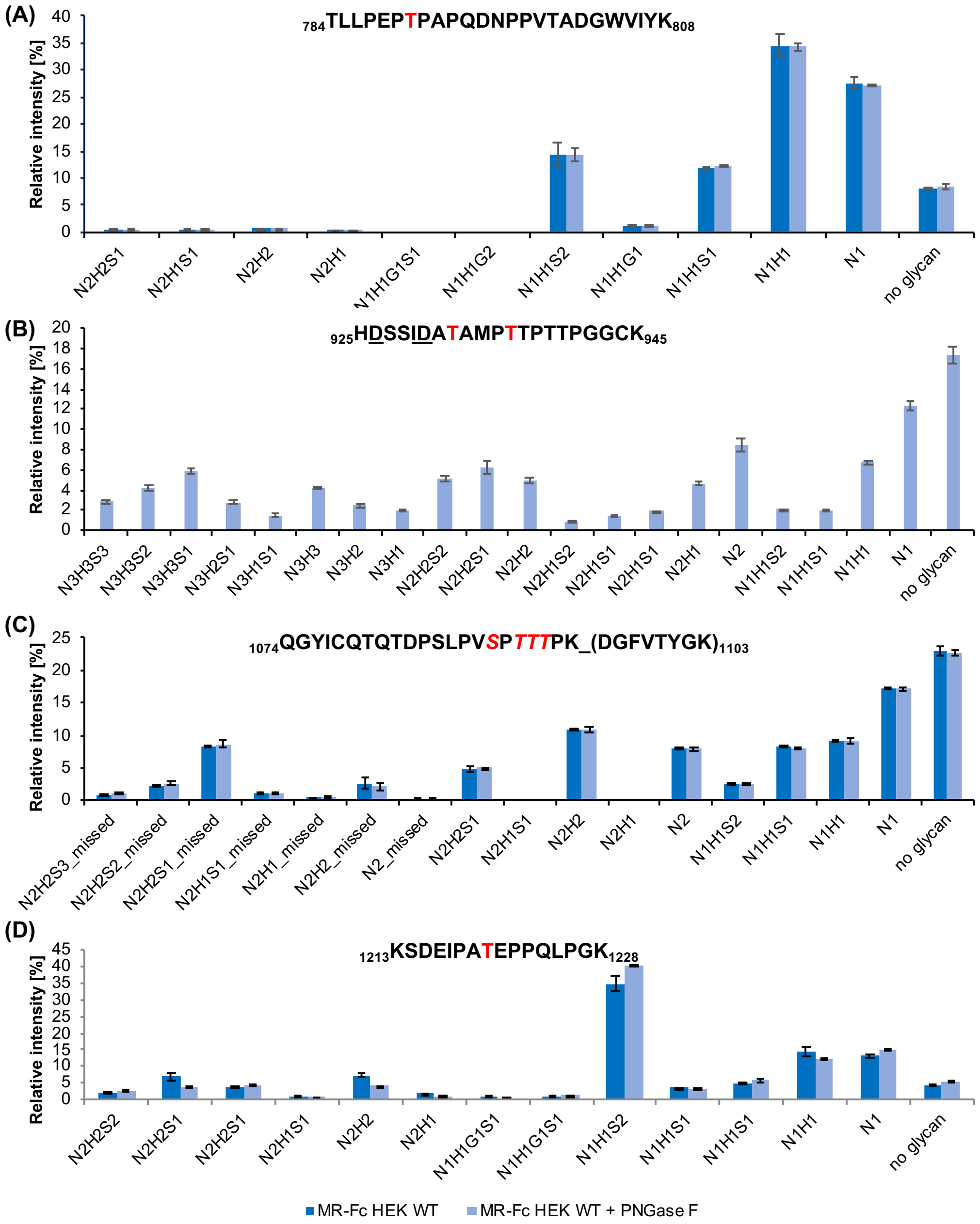

Figure S18: MR-Fc$_{HEK\ WT}$ O-glycopeptide relative quantitation. MR-Fc was treated with PNGase F and mock-treated prior in-gel trypsin digestion. D indicated deamidation after PNGase N-glycan release. Relative quantitation of all glycopeptides was performed in an automated manner as described previously [2]. The glycopeptide reference list contained all glycopeptides that were identified based on MS2 fragmentation but also lower abundant glycopeptides based on their exact mass, corresponding retention time, isotopic pattern and biosynthetic related glycan composition. Relative intensities were determined based on triplicate analysis and standard deviation is calculated. **S/T** is confirmed O-glycosylation site. ***S/T*** is O-glycosylation site region. N – N-acetylglucosamine, H – Hexose, F – Fucose, S – N-acetylneuraminic acid., G – N-glycolylneuraminic acid. +/- 1 standard deviation is shown.

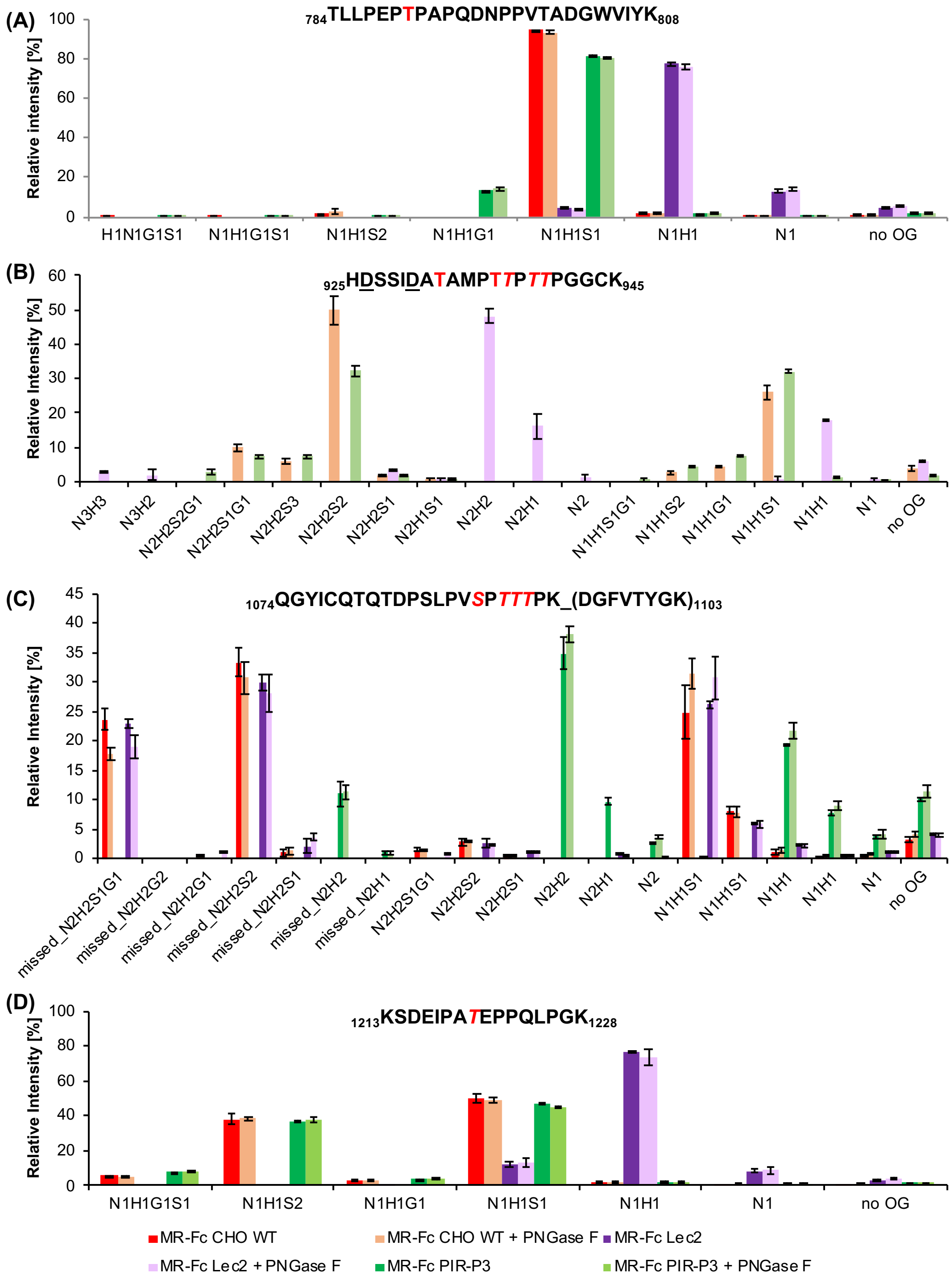

Figure S19: MR-Fc$_{CHO\ WT}$, MR-Fc$_{Lec2}$ and MR-Fc$_{PIR-P3}$ O-glycopeptide relative quantitation. MR-Fc was treated with PNGase F and mock-treated prior in-gel trypsin digestion. D indicated deamidation after PNGase N-glycan release. Relative quantitation of all glycopeptides was performed in an automated manner as described previously [2]. The glycopeptide reference list contained all glycopeptides that were identified based on MS2 fragmentation but also lower abundant glycopeptides based on their exact mass, corresponding retention time, isotopic pattern and biosynthetic related glycan composition. Relative intensities were determined based on triplicate analysis and standard deviation is calculated. **S/T** is confirmed O-glycosylation site. ***S/T*** is O-glycosylation site region. N – N-acetylglucosamine, H – Hexose, F – Fucose, S – N-acetylneuraminic acid., G – N-glycolylneuraminic acid. +/- 1 standard deviation is shown.

References

[1] Zeng, J., Eljalby, M., Aryal, R. P., Lehoux, S., Stavenhagen, K., Kudelka, M. R., Wang, Y., Wang, J., Ju, T., von Andrian, U. H., and Cummings, R. D. (2020) Cosmc controls B cell homing. *Nat Commun* **11**, 3990.

[2] Jansen, B. C., Falck, D., de Haan, N., Hipgrave Ederveen, A. L., Razdorov, G., Lauc, G., and Wuhrer, M. (2016) LaCyTools: A Targeted Liquid Chromatography-Mass Spectrometry Data Processing Package for Relative Quantitation of Glycopeptides. *J Proteome Res* **15**, 2198-2210.
